# Evaluation of zero counts to better understand the discrepancies between bulk and single-cell RNA-Seq platforms

**DOI:** 10.1016/j.csbj.2023.09.035

**Published:** 2023-09-29

**Authors:** Joanna Zyla, Anna Papiez, Jun Zhao, Rihao Qu, Xiaotong Li, Yuval Kluger, Joanna Polanska, Christos Hatzis, Lajos Pusztai, Michal Marczyk

**Affiliations:** a Department of Data Science and Engineering, Silesian University of Technology, Gliwice 44-100, Poland; b Computational Biology and Bioinformatics Program, Yale University, New Haven, CT 06510, USA; c Department of Pathology, Yale School of Medicine, Yale University, New Haven, CT 06510, USA; d Breast Medical Oncology, Yale Cancer Center, Yale School of Medicine, New Haven, CT 06520, USA; e Applied Mathematics Program, Yale University, New Haven, CT, USA

**Keywords:** Single-cell sequencing, Dropout rate, Zeros, Technical factors

## Abstract

Recent advances in sample preparation and sequencing technology have made it possible to profile the transcriptomes of individual cells using single-cell RNA sequencing (scRNA-Seq). Compared to bulk RNA-Seq data, single-cell data often contain a higher percentage of zero reads, mainly due to lower sequencing depth per cell, which affects mostly measurements of low-expression genes. However, discrepancies between platforms are observed regardless of expression level. Using four paired datasets with multiple samples each, we investigated technical and biological factors that can contribute to this expression shift. Using two separate machine learning models we found that, in addition to expression level, RNA integrity, gene or UTR3 length, and the number of transcripts potentially also influence the occurrence of zeros. These findings could enable the development of novel analytical methods for cross-platform expression shift correction. We also identified genes and biological pathways in our diverse datasets that consistently showed differences when assessed at the single cell versus bulk level to assist in interpreting analysis across transcriptomic platforms. At the gene level, 25 genes (0.12%) were found in all datasets as discordant, but at the pathway level, 7 pathways (2.02%) showed shared enrichment in discordant genes.

## Graphical Abstract

**Figure:**
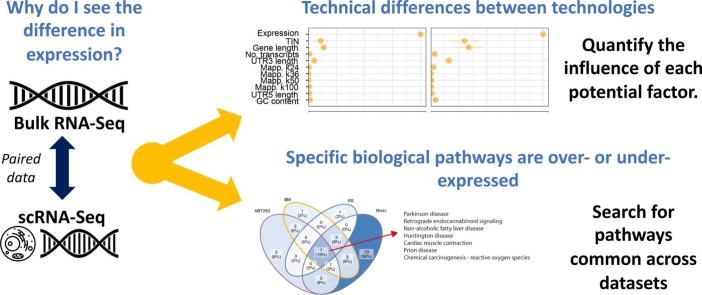

## 1 Introduction

RNA sequencing (RNA-Seq) is a commonly used technology in exploratory and translational research with great potential for clinical applications [1]. In typical bulk RNA-Seq experiments, millions of cells are mixed and then sequenced together, allowing researchers to measure the average gene expression signal representing the whole population of cells in a sample. In recent years, thanks to advances in sample preparation and sequencing technology itself, profiling of individual cells (single-cell RNA-Seq) also became possible [2]. By measuring the level of expression of many genes in many individual cells simultaneously, we can determine the transcriptomic profiles that characterize various cell types. However, the transcriptome is a dynamic structure that responds to a wide variety of external and internal factors that a cell is exposed to, and therefore expression heterogeneity is expected even in the same cell type under similar conditions [3]. Analyzing this heterogeneity using scRNA-Seq data allows for a deeper understanding of cellular states and their relationship to diseases and environmental factors [4]. Several platforms for scRNA-Seq have been developed, each with its advantages and disadvantages [2], [4], but two main types of technologies can be distinguished: (i) droplet-based protocols using unique molecular identifiers and 3' or 5' sequencing; (ii) protocols that measure the full-length transcript. The latest droplet technologies enable parallel profiling of hundreds of thousands of cells, which is much higher than what can be achieved with full-length protocols. In this work, the 10x Genomics Chromium droplet platform, which uses gel beads to capture single cells [5], was used to obtain gene expression data for individual cells.

The measurements obtained in single-cell transcriptomics are counts representing the number of sequencing reads mapped to a gene. In bulk sequencing, which yields combined signals from many cells, and therefore, lower variability in expression value, data analysis is much easier than in scRNA-Seq. The counts in scRNA-Seq are also naturally more variable due to inter-cell heterogeneity, mixtures of cell types, and stochastic bursts of expression, among others [6]. Additional challenges in the computational analysis of single-cell counts include high dimensionality of the data, greater measurement noise, and lower detection limits [7], [8]. A well-known feature of scRNA-Seq is data sparsity, i.e. a high percentage of zero reads (so-called dropout rate) [9]. While the proportion of zeros in bulk RNA-Seq data usually ranges between 10% and 40%, in scRNA-Seq, the proportion can be as high as 90% for selected cells [9]. Some null expressions may reflect a true lack of biological expression, where the gene is simply not expressed in the cell or may be due to the stochasticity of gene expression. These genes called true biological zeros are preserved at zero expression levels by some imputation methods [10], [11], but the other replace all zeros in the dataset [12]. Zeros can also occur even when the transcript is expressed in the cell but is completely undetected in its mRNA profile [13]. The reason for this type of zeros may be too low sequencing depth, inefficient cDNA polymerization, or the occurrence of various amplification errors. Other factors affecting the level of zeros include differences in the composition of nucleotides, the length of the tested RNA fragments, and differences resulting from the variability of the degradation rate of tested molecules [14]. Examining and understanding the statistical properties of cells with zero values can help facilitate the development of best practices for various data analytic tasks in the field [15].

While the problem of the existence of an excessive number of zeros in scRNA-Seq has been studied well [9], [13], [15], [16], less is known about the causes of other, more general discrepancies in gene expression that are observed between two platforms: bulk and single-cell. Also, in the literature, it is explained that the main source of zeros in scRNA-Seq is due to too low sequencing depth, thus low expressed genes could not be captured, but other possible sources are not evaluated. The main goal of the presented research was to quantify and identify sources of differences in expression between bulk and scRNA-Seq data, particularly concerning genes with low expression levels. For this purpose, four publicly available datasets, where paired bulk RNA-Seq and scRNA-Seq data were available from the same platform, were analyzed. We investigated several factors that could potentially drive the expression shift. First, we identified which factors influence the observed dropout rate the most using a machine learning-based approach. Next, we searched for the genes that show consistent deviations from expression across different datasets. Finally, we determined a group of biologically related genes with cross-platform differences that could lead to false-positive discoveries at the scRNA-Seq level in comparison to other omics findings.

## 2 Material and methods

### 2.1 Datasets

In the presented study, 4 different datasets were used (Table 1). Each dataset consists of paired transcriptomic profiles measured on bulk and single-cell levels. The first dataset, labeled as ABT263, refers to experiments that involved repeated applications of cytotoxic agents to the in vitro cultures of human triple-negative breast cancer cell line MDAMB-468 [17]. The measurements for two parallel sets of identical experiments (biological replicates a and b) at baseline and after treatment, were investigated. In total, 4 different samples and 4680 cells were sequenced (on average 1170 cells per sample). The second dataset (RH41) includes measurements from the human alveolar rhabdomyosarcoma cell line Rh41. The unsorted cells were extracted from a large experiment [18], [19], but only a single sample was analyzed with 7004 cells sequenced. The third dataset includes bulk RNA-Seq and droplet-based scRNA-Seq of human bone marrow mononuclear cells (BM) for 8 different patients who were treated as individual samples [20]. In total 29466 cells were sequenced (on average 3683 cells per sample). The last dataset, labeled as PR, investigated prostate specimens from 3 young male organ donors aged 18–31 registered at Southwest Transplant Alliance. From those donors' a variety of tissues was sequenced. In this study, 4 different tissues, here treated as samples, were used as they feature matched single cell and bulk results (BE basal epithelium, LE luminal epithelia, OS other epithelia, FMSt fibromuscular stroma) [21]. In total 48517 cells were sequenced (on average 12143 cells per sample). A detailed number of sequenced cells in each sample can be found in Supplementary Table 1.

**Table 1:** Summary of a used dataset in the investigation of the dropout effect.

Data set	Accession no.	Tissue or cell line	Samples	Disease	Bulk RNA-Seq platform	scRNA-Seq platform	Total cell no.
ABT263	PRJNA657088	MDAMB-468	4	Cancer	Illumina HiSeq 4000	10x Genomics Chromium v2	4680
BM	GSE120444	Bone Marrow	8	Healthy	Illumina HiSeq 3000	10x Genomics Chromium v2	29466
PR	GSE120716	Prostate	4	Healthy	Illumina NextSeq 500	10x Genomics Chromium v3	48517
RH41	GSE113660	Rh41	1	Cancer	Illumina HiSeq 4000	10x Genomics Chromium v2	7004

### 2.2 Data pre-processing

All datasets were preprocessed in the same way to ensure comparability of the results. Initially, the bulk and single-cell data were merged by projecting to the same transcriptome according to the ENSEMBL release 84 annotations. In total, 19797 protein-coding genes were included in the analysis. The next step was to create pseudo-bulk (cumulative scRNA-Seq) samples based on the scRNA-Seq data. For this purpose, the numbers of counts for each gene were added up from all cells per sample. In the RH41 cell line experiment, the three single-cell replicates were combined by adding up the cell counts to form a single pseudo sample. In the BM experiment, for the patient labeled “C” in the single-cell experiment, the first technical replicate was selected. Finally, to compare the expression rates in the experiments, normalization was carried out by calculating counts per million statistics using the *edgeR* package function with the log2 transformation [22]. Dropout rates for every gene were calculated for the scRNA-Seq data as the proportion of cells with zero counts to the total number of cells in a sample. To investigate potential batches between samples, the tSNE (t-Distributed Stochastic Neighbor Embedding) [23] projections were created on the scRNA-Seq platform in each study separately. P-value from guided PCA using the *BatchI* R package was calculated to quantify the batch effect [24]. A threshold to divide genes into low- and high-expression groups was found with *GaMRed* software [25] on bulk expression data. Downsampling of scRNA-Seq data was done using the *downsampleReads()* function from *DropletUtils* R package [26] which generates a UMI count matrix after downsampling of raw sequencing data from the molecule information file produced by CellRanger software [5].

### 2.3 Technical factors description

To catch possible technical factors related to differences in gene expression between bulk RNA-Seq and scRNA-Seq, the range of characteristics for 19797 protein-coding genes was collected. The first batch of factors was retrieved from the ENSEMBL database [27]. In this group, the factors can be divided into two types: (i) single gene information; (ii) information from averaging of multiple factors. In the first type, there is GC content of the gene sequence, gene length, and the number of transcripts per gene. Moreover, for the dominant transcript (the one with the highest Transcript Support Level) of each gene the CDS, UTR3’, UTR5’, and transcript length were collected. The second type consists of the mean length of CDS, UTR3’, UTR5, and transcript length for all transcripts per gene. Next, the mappability was calculated as the percentage and count of gene coverage by single-reads mapped to the genomic region which is a fraction of that region that overlaps with at least one unique k-mer (k equal 24, 36, 50, and 100) [28]. In total, 19 characteristics were collected. The next factor was calculated separately for each sample using bulk expression data. The Transcript Integrity Score (TIN) [29] represents the non-uniformity of the sequence coverage for each transcript measured by Shannon’s entropy. TIN varies from 0 to 100, where 100 means perfect RNA integrity. For each gene, the median TIN from all transcripts that belong to this gene was calculated. Finally, the gene expression from bulk RNA-Seq representing true gene expression level was included in the investigation as the last technical factor.

### 2.4 Technical factors analysis methods

For collected measures, both Pearson and Spearman rank correlations were calculated, and highly correlated factors were removed. Before modeling, missing values were imputed using the k-nearest neighbor method, then centered and scaled. Next, two regression models: the eXtreme Gradient Boosting (XGBOOST) and Bayesian Regularized Neural Networks (BRNN), were fitted for each sample within a dataset. The model was built to predict the dropout rate based on collected factors with the following formula:

(1) Dropoutrate∼Expression+TIN+Genelength+No.transcripts+UTR3length+Mapp.k24+Mapp.k36+Mapp.k50+Mapp.k100+UTR5length+GCcontent

The model was built using the original scale of each factor and binned data (5 bins for each factor). The variable importance of each factor with its 95% confidence interval (CI) for the mean in each dataset separately was calculated using the *caret* R package [30]. XGBOOST and BRNN were run using the *train()* function from the *caret* package with default parameters including hyperparameter tuning with bootstrapping (25 repetitions), grid search method, and minimizing root-mean-squared error (RMSE). For XGBOOST seven parameters were tuned: *nrounds* (no. of boosting iterations), *max_depth* (max. tree depth), *eta* (shrinkage), *gamma* (minimum loss reduction), *colsample_bytree* (subsample ratio of columns), *min_child_weight* (minimum sum of instance weight), and *subsample* (subsample percentage). For BRNN the number of neurons in each hidden layer was changed from 1 to 3. Since the goal was to obtain only the importance of each technical factor, models were built using all data without feature selection. The performance of model fit was measured using R^2^, RMSE, and mean absolute error (MAE). The above-described process of variable importance estimation was repeated for the BM dataset by excluding different cell types. Specifically, cell types labeling was performed by *SingleR* framework [31] with Human Primary Cell Atlas Data (HPCAD) [32] from *celldex* R package. Finally, each cell type was removed one at a time and both XGBOOST and BRNN were run on binned and original data.

### 2.5 Non-linear regression model

To estimate the relationship between the dropout rate measured on a single-cell level and gene expression measured on a bulk level, a non-linear regression model with a five-parameter logistic curve was fitted (5PL) using the *drc* R package [33]. The model is represented by the following formula (Eq. 2):

(2) $$f\left(x\right)=c+\frac{d-c}{{\left(1+\exp\left(b(\log\left(x\right)-e)\right)\right)}^{f}}$$

where *d* is the higher asymptote (fixed at 1), *c* is the lower asymptote (fixed at 0), *e* is the value halfway between the asymptotes of *d* and *c* (bounded between minimum and maximum values of normalized expression), *b* is the slope at the inflection point and *f* represents the model symmetry (the function is asymmetric for *f* different from 1). The parameters of the model were estimated by analysis of the model residuals. Specifically, the Kolmogorov-Smirnov test was run on model residuals, excluding genes with a dropout rate equal to 0 or 1, and the *D* statistic was calculated. The best parameters of the model were set as the ones that minimize *D*. The analysis was run on each sample within each dataset.

### 2.6 Regression model residuals analysis

For each dataset and sample, residuals from the non-linear regression models were centered and scaled. Genes were marked as over-expressed if scaled residuals were higher than 2 and under-expressed if scaled residuals were lower than −2 only in bulk experiments but not in single-cell data.

An ordered list of genes according to their value of residual from the non-linear regression model was used as the input to Gene Set Enrichment Analysis (GSEA) [34]. Enrichment of 347 KEGG pathways [35] was tested using GSEA implementation in the *fgsea* R package [36] with the gene permutation parameter. A normalized enrichment score (NES) was used as a measure of each gene set’s effect size. The positive value of NES will correspond to genes with a positive value of residuals and inversely for the negative values. The pathway was claimed to be significantly enriched when the adjusted p-value after Benjamini-Hochberg multiple testing correction was lower than 0.05. Significant pathways were marked as over-expressed if NES was higher than 0 and under-expressed if NES was lower than 0 only in bulk experiments but not in single-cell data.

### 2.7 Differential analysis

To find the differences in biological findings between platforms, the ABT263 dataset was used. The differentially expressed genes (DEGs) were established by comparison of samples 1a and 1b with samples 2a and 2b (baseline vs 3 days after navitoclax treatment) using *DESeq2* [37] package separately for bulk and scRNA-Seq data. Functional analysis was performed on KEGG pathways using GSEA implementation in the *fgsea* R package [36] with the DESeq2 test statistic used as the gene rank.

## 3 Results

### 3.1 Identification of batch effects

As four datasets with several samples were included (Table 1, Supplementary Table 1), we first performed tSNE projection to reveal any batch effects that could be potentially introduced during the preparation of scRNA-Seq experiments (Supplementary Figure 1). In the ABT263 study, where two biological replicates are present, samples are well-mixed, showing no technical differences between cells. We observed the same for the BM dataset, which was correctly separated by cell subtypes, not by samples. Those findings were supported by the results from the BatchI algorithm (p-value>0.05, Supplementary Table 2). In the PR dataset, we can observe clusters of cells from the same sample and clusters of mixed cell types, which indicates that the batch effect of sample donors may be present. Nevertheless, donor information is not available for the PR dataset, so the observed differences are due to biological factors (p-value=0.0079, Supplementary Table 2), which is an expected phenomenon. Thus, in further analysis, each sample (cell type in PR) is analyzed separately, so the batch has a negligible effect on results and conclusions.

### 3.2 Characterization of discrepancies between platforms

**Fig. 1:**
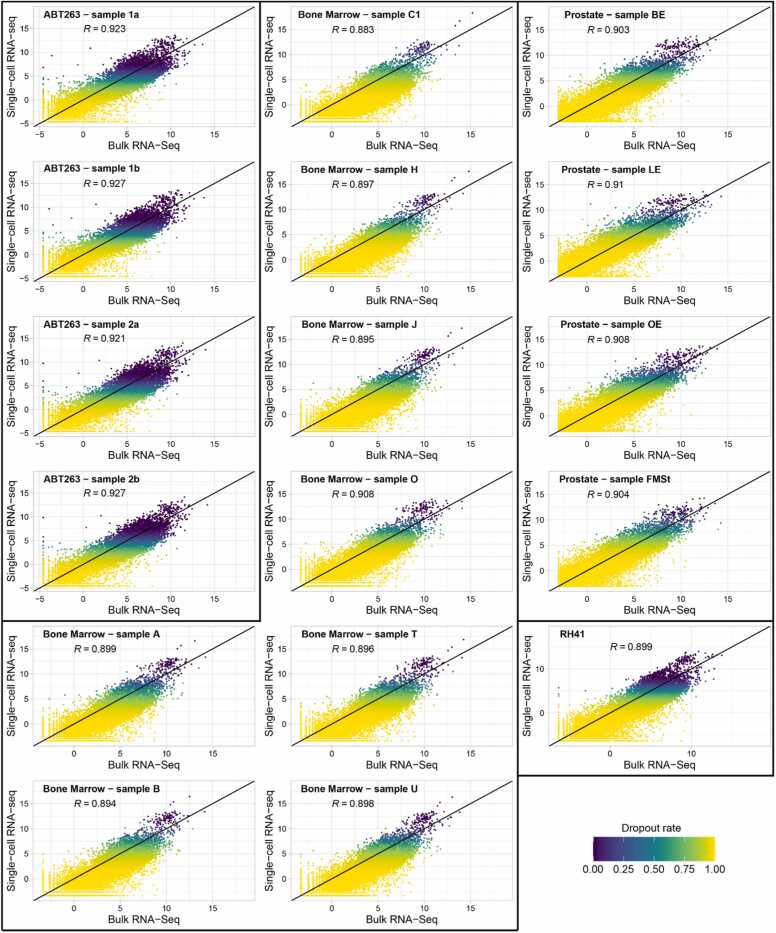
Comparison of expression of individual genes between bulk RNA-Seq and summarized scRNA-Seq for all analyzed samples. Both the x- and y-axis represent normalized expression within each platform. The color scale shows the dropout rate (proportion of zero counts per gene) from blue (low) to yellow (high). R coefficient refers to Spearman rank correlation.

We created summarized scRNA-Seq samples by adding counts from each cell measured in a particular sample. We checked the relation between summarized counts from scRNA-Seq and bulk RNA-Seq for each of the 19797 protein-coding genes present on the analyzed platform (Fig. 1) and calculated the dropout rate (proportion of zero counts per gene) within each sample using scRNA-Seq data. Since the aim of the study was to identify aspects that influence dropout rates using single-cell and bulk sequencing platforms, we do not introduce any corrections to the data (e.g., for gene length) when comparing expression levels, to determine the significance of this factor. For each sample, we excluded the genes with zero counts in both bulk and single-cell data, so the dropout genes were those that were detected in bulk sequencing and not in scRNA-Seq. The number of dropout genes varies between datasets and samples from 580 (2.93%) to 1742 (8.8%, Supplementary Table 3). Despite strong correlations of overall gene expression between both platforms (Fig. 1), we observe a pattern of dropout rate; dropout is stronger but highly variable for genes with lower expression. This pattern is consistent across samples and datasets, however, in ABT263 and RH41, there is a much lower dropout rate for highly expressed genes compared to BM and PR datasets. It can be noticed that in individual case's dropout rate equal to 1 is observed even for genes highly expressed in bulk sequencing (Fig. 1). Finally, we calculated the relative log expression (RLE) for bulk and scRNA-Seq samples (Supplementary Figure 2). It can be observed that regardless of data normalization, scRNA-Seq has on average lower relative expression compared to bulk in all analyzed samples and datasets.

**Fig. 2:**
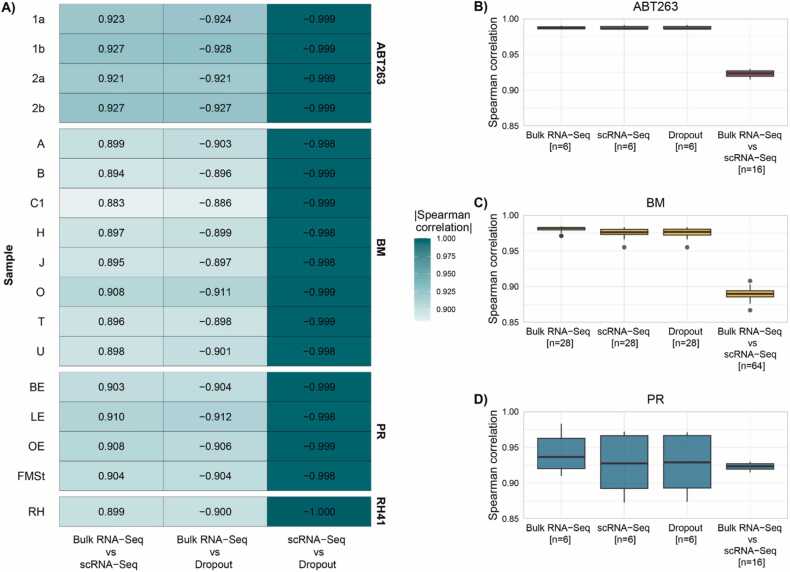
Spearman rank correlation for pairwise comparisons of different platforms and dropout rate. A) Correlation within each sample for a particular platform. B), C), and D) show correlation coefficients between different samples grouped within each dataset, dropout rate, and between scRNA-Seq and bulk RNA-Seq.

Using Spearman rank correlation, we quantified the relationship between bulk RNA-Seq gene expression, summarized scRNA-Seq gene expression, and dropout rate (Fig. 2). Calculations were performed within and between samples in each dataset separately. High gene expression observed in scRNA-Seq corresponds to high expression in bulk RNA-Seq, and lower gene expression is related to a higher dropout rate (Fig. 2A). The highest correlation is observed between gene expression in scRNA-Seq and dropout rate. To estimate the divergence in observed signal values, we calculated the correlation between different samples within each dataset, dropout rate, and between scRNA-Seq and bulk RNA-Seq (Figure 2BCD). In two datasets, ABT263 and BM, we observe a strong positive correlation between the samples measured on the same platform, and for the dropout rate between samples. The correlation between bulk RNA-Seq and scRNA-Seq is significantly lower (ANOVA p-value<0.0001 for both datasets) compared to the correlations within each platform (Supplementary Table 4). Thus, there are additional discrepancies when comparing gene expression between bulk RNA-Seq and scRNA-Seq. In the PR dataset, where multiple donors are grouped into specific tissue types, differences in correlations are not so obvious (variability within the platform due to different donors is similar to variability between platforms).

To explain the differences in results between datasets, we compared selected characteristics (Supplementary Figure 3ABC). The percent of non-expressed genes was higher in ABT263 and BM than in PR (∼23% vs ∼15%). The sequencing depth of samples from the ABT263 set was more than twice that in others (∼50 M reads vs ∼20 M reads). Also, the mean dropout rate per gene in scRNA-Seq samples was much lower in ABT263 (∼0.72 vs ∼0.93).

Next, we investigated the influence of sequencing depth on differences between platforms by downsampling the scRNA-seq data from the ABT263 set (Supplementary Figure 3D). Reducing the number of sequencing reads by half did not significantly change the correlations when comparing samples within the scRNA-Seq platform or between platforms. Even when the proportion of library size after downsampling was equal to 0.1, the correlation was only slightly decreased (from 0.986 to 0.982 on average when comparing samples within the scRNA-Seq platform and from 0.923 to 0.919 on average when comparing samples between platforms).

In some experiments, researchers filter out the group of low-expression genes prior to statistical analysis. To mimic this situation, for each sample, we separated low- and high-expression genes using bulk expression data (example in Supplementary Figure 4A). This resulted in lower correlations between platforms and between bulk expression and dropout rate than when all genes were taken (Supplementary Figure 4BC). As expected, in a group of low-expression genes the correlation between platforms was much smaller than in a group of high-expression genes in all samples. The differences between groups of genes were the biggest in the BM dataset.

### 3.3 Definition of influential technical factors for model building

**Fig. 3:**
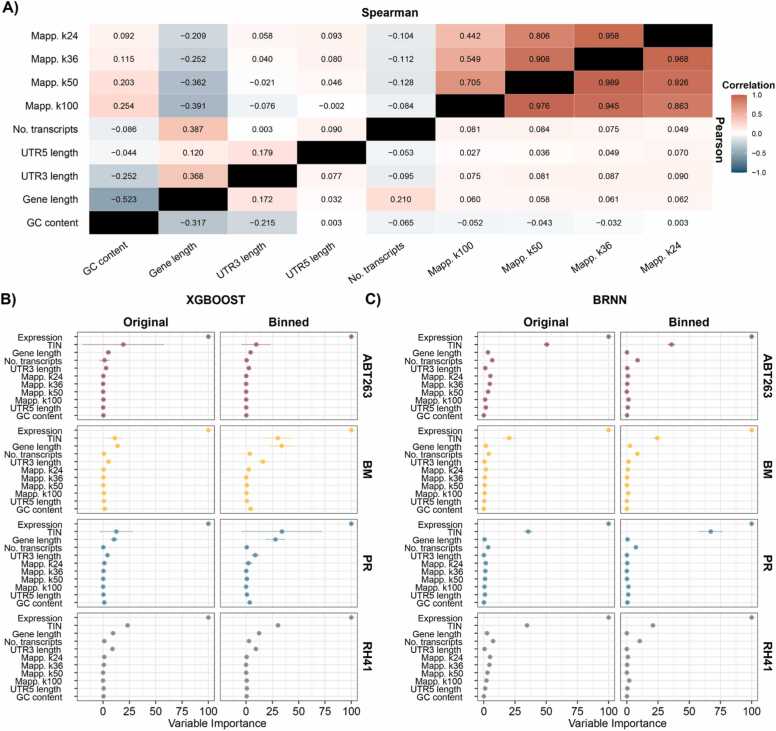
Investigation of different technical factors for dropout rate prediction. A) Correlation between nine selected factors for all investigated genes. B) and C) show variable importance from XGBOOST and BRNN models respectively, built on all factors with 95% CI for the mean (calculated from all samples within the study).

We collected nineteen different technical parameters that could potentially influence the value of gene expression observed on a single-cell level for each of the 19797 protein-coding genes from multiple bioinformatic databases (see Methods). Since we observed the high correlation between factors that could potentially lead to redundancy of information during the construction of the prediction model (Supplementary Figure 5), some of these variables were filtered out. For the mappability, we considered further only variables in the percentage scale. Since mean UTR3’, UTR5’, CDS, and transcript length are highly correlated with the same factors calculated only for the gene-dominant transcript (see Methods), we removed them. Finally, CDS and transcript length are highly correlated with gene length, so we used only the gene length in model building. After the removal of redundant variables, we left 9 technical factors in the analysis. Even though the mappability scores calculated for different k-mers were highly correlated to each other (Spearman correlation range from 0.442 to 0.958), they showed slightly different skewed distributions, and therefore, we kept them. Comparison of other factors yielded a lower magnitude of correlation coefficient (0.5 or lower, Fig. 3A), the highest among these was between gene length and GC content, as well as between gene length and the number of transcripts. Additionally, we added two more variables to model building: TIN [23], which represents the quality of the transcript (if not degraded and with uniform coverage across the transcript), and gene expression measured on a bulk level, representing the number of mRNA molecules in the sample.

### 3.4 Machine learning for technical factor importance estimation

To find the most important, non-redundant factors responsible for discrepancies in gene expression between platforms we created two different regression models. Since the dropout rate is almost perfectly negatively correlated with cumulative gene expression from scRNA-Seq, we used it as a dependent variable in the model. The benefit of using dropout is that it always ranges between 0 and 1 and its meaning is consistent across different datasets and samples, while the normalized scRNA-Seq expression level may vary. The selected 11 technical factors including bulk-level gene expression, that capture unique attributes of protein-coding genes, were set as independent variables in the model.

We used two different nonlinear machine learning (ML) techniques to construct a dropout rate prediction model: (i) XGBOOST; (ii) BRNN. To reduce noise in the data, we also performed data binning (5 bins per factor). We used both original and binned measurements to construct a model on each sample and calculated the importance of each variable in the model. Next, we averaged the importance metrics of each variable between samples to show the mean effect in each dataset (Figure 3BC). The models were better fitted to the data on the original scale (R^2^ from 0.668 to 0.838 for BRNN and from 0.743 to 0.869 for XGBOOST) than to binned data (R^2^ from 0.494 to 0.798 for BRNN and from 0.556 to 0.804 for XGBOOST; Supplementary Table 6). The original and binned data gave similar results, and the main difference was observed between ML techniques since XGBOOST makes use of slightly more features (technical factors) to predict the model output than BRNN. Regardless of the dataset and ML technique used, the main variables that influenced the dropout rate were bulk gene expression and TIN. Both factors were highly correlated with the dropout rate in each sample (Fig. 2A, Supplementary Figure 6). We also identified other factors with smaller importance, including gene length, number of transcripts, and UTR3’ length. The mappability (all k-mers), GC content, and UTR5’ length did not correspond to the dropout rate level in our ML models.

Next, we investigated if the obtained ranking of important technical factors is not specific to certain cell types that are sensitive to the scRNA-seq protocol. The BM dataset is derived from a complex tissue, so we used it and annotated each cell with Human Primary Cell Atlas Data. We found 13 different cell types (Supplementary Figure 7). We do not have the bulk expression data for each cell type in the BM dataset, so a direct comparison between platforms was not possible. Instead, we removed one cell type at a time from scRNA-Seq data, estimated the dropout rate, and fitted two ML models as previously described (Supplementary Figure 8). In all cases, bulk gene expression was the most important factor in the model. When T-cells were excluded, we observed a higher importance of TIN and a lower importance of gene or UTR3’ length, especially when the BRNN model was used. For other cell types, no consistent anomalies in variable importance were found.

### 3.5 Differences in biological effects between platforms

**Fig. 4:**
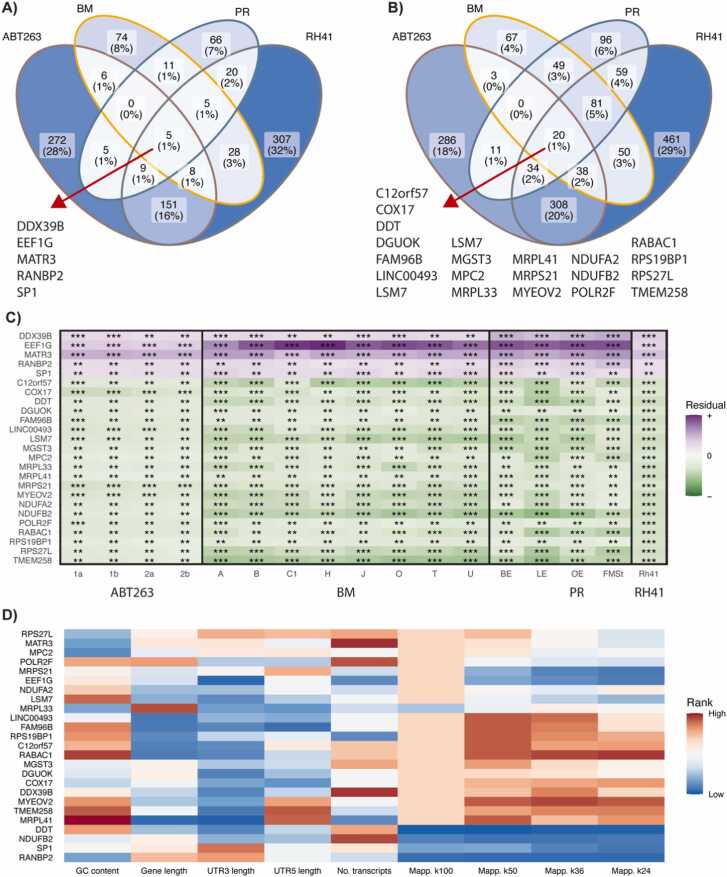
Results of gene-level analysis using residuals of the 5PL model. A) Venn diagram of genes with scaled residuals higher than 2 and their coverage across datasets. B) Venn diagram of genes with scaled residuals lower than −2 and their coverage across datasets. C) Heatmap of scaled residuals for genes marked in panels A and B. Stars represent the magnitude of scaled residuals: ** |scaled residual| > 2, ***|scaled residual| > 3. D) Heatmap of technical factors’ ranks for genes marked in panels A and B.

We searched for the genes that show consistent deviations in expression between platforms across our datasets. At first, we fitted a five-parameter logistic model to find the pattern of relationships between gene expression measured on bulk RNA-Seq and dropout rate calculated for scRNA-Seq data in each sample (Supplementary Figure 9) and calculated the residuals from the model (Supplementary Figure 10). The residual value higher than zero (over-expressed genes) represents larger expression on bulk level compared to single-cell level (thus potentially increased dropout rate), while the opposite (under-expressed genes) is indicated by residuals lower than zero. The number of genes with much higher or lower residuals (absolute value of scaled residual higher than 2) was similar between samples, ranging from 4.9% to 8.1% (Supplementary Table 5). For each dataset, we selected common over- or under-expressed genes and compared them between datasets. For over-expressed genes, only 5 were common (Fig. 4A), while for under-expressed genes 20 were common (Fig. 4B). For these genes, the absolute value of the scaled residual was higher in BM and PR datasets (Fig. 4C). We also checked the values of 9 technical factors analyzed above for these common genes (Fig. 4D). We found higher GC content for MRPL41 and RABAC1 or a higher number of transcripts for DDX39B and MATR3, but we found no consistent pattern.

**Fig. 5:**
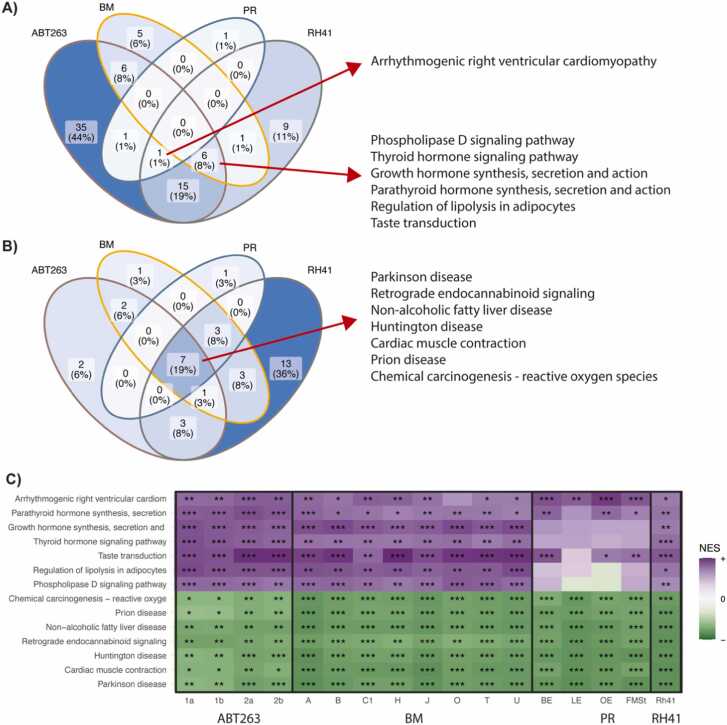
Results of KEGG pathways analysis with GSEA algorithm using residuals of the 5PL model. A) Venn diagram of significantly over-expressed pathways and their coverage across datasets. B) Venn diagram of significantly under-expressed pathways and their coverage across datasets. C) Heatmap of NES value from GSEA analysis for pathways marked in panels A) and B). The intensity of the purple color represents the NES value for over-expressed pathways, while green is for under-expressed. Stars represent the significance of NES: * FDR < 0.05, ** FDR < 0.01, *** FDR < 0.001.

Next, we searched for groups of biologically related genes that showed similar patterns of discrepancies in gene expression between single-cell and bulk RNA-Seq. The residual values from the 5PL model were used as gene ranks in GSEA using KEGG pathways. For different datasets and samples, we found a different number of pathways showing over-expression (higher values of residuals for genes within pathways) or under-expression (Supplementary Figure 11, and Supplementary Figure 12, respectively). Next, the common pathways across all datasets were examined to investigate if the genes with a discordant expression between platforms correspond to common biological processes regardless of the investigated phenotype (Fig. 5). For pathways showing over-expression in GSEA, none were common in all datasets (Fig. 5A), but 7 were shared among 3 of the 4 datasets. For pathways showing under-expression in GSEA, 7 were shared by all datasets (Fig. 5B). The level of significance of pathway alteration by GSEA was also higher for under-expressed pathways (Fig. 5C). As an example, we illustrated the relationship between gene expression measured in bulk and summarized single-cell levels for genes in “Parathyroid hormone synthesis, secretion and action” pathway (Supplementary Figure 13), where a consistent decrease of gene expression in scRNA-Seq is observed. Similar patterns are observed for the other under-expressed pathways.

Out of the highlighted pathways the “Chemical carcinogenesis - reactive oxygen species” can be distinguished. The reactive oxygen species (ROS) are well known to link with DNA damage process. Also, oxidative stress may be involved in mediating the degradation of RNAs, which is an important technical factor that influences sequencing results [38]. Several hormonal pathways are also observed. The mRNA decay rates are influenced by the cellular microenvironment as well as hormones [39]. Other common pathways are linked to heart and brain diseases, which are loosely connected to datasets under study. Thus, we tested the hypothesis that these pathways might be correlated with technical factors described in the previous paragraph. For each pathway, we selected its genes, and by using the Mann-Whitney test and rank-biserial correlation effect size measure we compared if any value of technical factor for these genes is not higher or lower than for other genes (Supplementary Figure 14; Supplementary Table 7). Ten out of fourteen pathways were correlated with bulk expression and three with TIN, which were the most important factors influencing the shift between platforms. Some pathways were also correlated with gene length or UTR3 length. Only 1 pathway, “Regulation of lipolysis in adipocytes” was not connected with any technical factor.

**Fig. 6:**
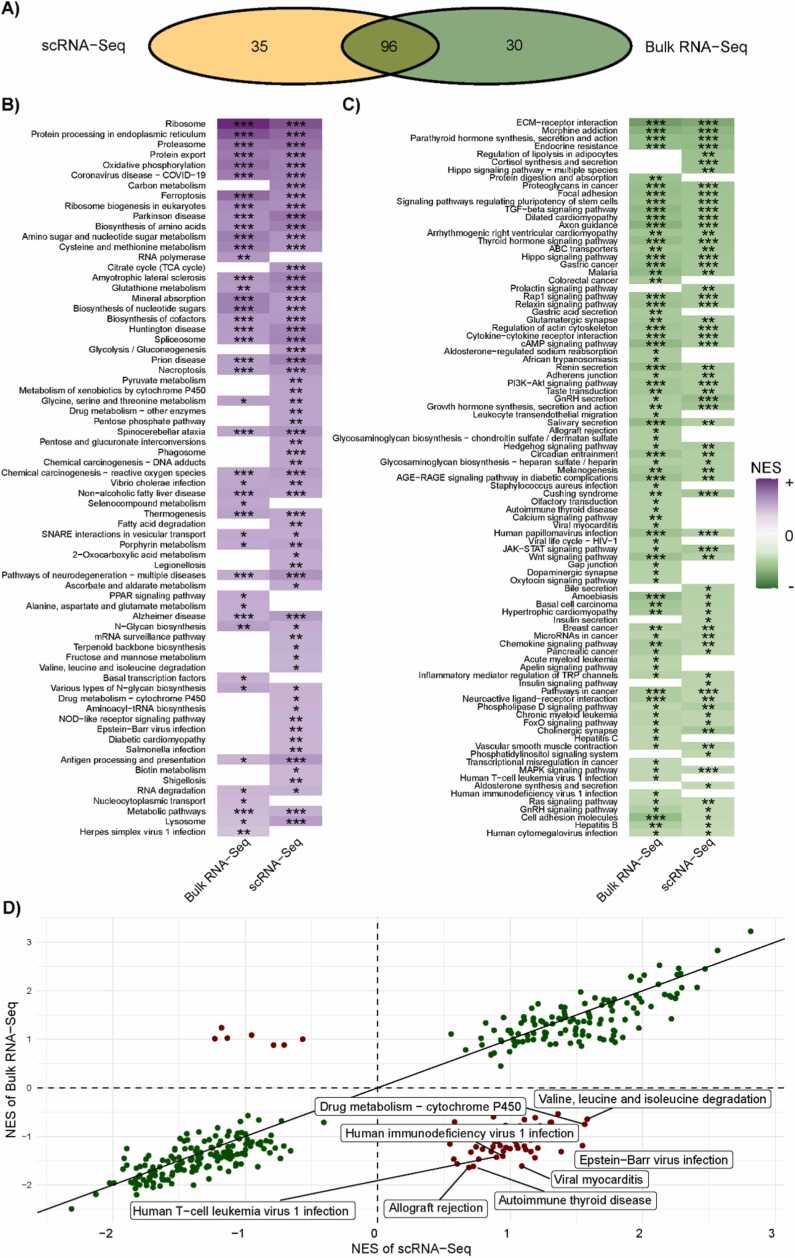
Results of KEGG pathways analysis with GSEA algorithm for ABT263 dataset between biological conditions (baseline vs 3-days of navitoclax treatment. A) Venn diagram showing significantly over-expressed pathways and their coverage across platforms. B) and C) Heatmap of NES value from GSEA analysis for significant pathways in at least one platform. The intensity of the purple color represents the NES value for over-expressed pathways, while green is for under-expressed. Stars represent the significance of NES: * FDR < 0.05, ** FDR < 0.01, *** FDR < 0.001. Panel D) shows the NES value from GSEA analysis on both platforms. The green color shows the pathways with the same direction of expression change, while the red color represents the opposite response. Pathways with given names was marked as significant (FDR < 0.05) in at least one platform and showed different direction of response to treatment.

We also tested another approach to find differences in biological effects between platforms. Using the ABT263 dataset, we run differential gene expression analyses and functional analyses comparing samples before and after navitoclax treatment (Fig. 6). Overexpressed pathways specific for the bulk RNA-Seq data included different cancer pathways where navitoclax is used for treatment: Acute myeloid leukemia [40], Colorectal cancer [41], and Human T-cell leukemia virus 1 infection [42]. Other pathways were related to cellular processes activated by inflammation and their response to ABT263. The Allograft rejection pathway is affected by navitoclax in the case of organ transplant procedures through pre-treatment that significantly improves 28 days post kidney transplantation [43]. Navitoclax being a BCL2 protein inhibitor involves pathways such as Calcium signaling [44], Nucleocytoplasmic transport [45], and RNA polymerase [46]. In the case of pathways overexpressed in the scRNA-Seq data, we observe metabolic processes activated by navitoclax. Among these pathways, there are Pyruvate metabolism [47], Phagosome [48], Pentose phosphate pathway [49], Fatty acid degradation [50], and Citrate cycle [51]. Drug metabolism pathways found that are influenced by ABT-263 are cytochrome P450 [52] and other enzymes [53]. Finally, processes linked to the secretion of insulin [54] and bile [55] were found as altered by navitoclax.

## 4 Discussion

We performed a comprehensive evaluation of gene expression differences between single-cell and bulk RNA-Seq platforms using 4 diverse datasets. In general, differences between biological/technical replicates were minimal across all platforms and the differences between platforms were greater than comparing replicates on the same platform. Analysis of tSNE plots revealed a small potential batch effect in the PR dataset, for which we also found smaller correlations between samples measured on the same platform than in the other sets. For each sample, we found multiple genes with no counts (zeros) in all cells on the single-cell platform, but with medium or high expression observed on a bulk level. Also, there were many genes with a higher or lower expression at the single-cell level in comparison to bulk, this was seen mostly among the low-expression regions of the data.

We examined if low mRNA expression is the only reason for differences between platforms as suggested by earlier literature [9], [13], [16]. We tested various technical factors that represent many potential sources of bias: (i) gene expression level related to the phenotype under study; (ii) TIN exhibiting relation to problems with sample storage and preparation; (iii) gene length, UTR3’ length, and no. of transcripts representing the construction of the gene; (iv) mappability denoting problems during alignment to the transcriptome. We found that the most important factor is the level of gene expression, however, TIN, gene length, and UTR3 length were also identified as non-redundant independent variables that influence the dropout rate in our model. This indicates that the architecture of a gene or not uniform mapping of mRNA short sequences along the transcript are also important sources of expression discrepancies between scRNA-Seq and bulk RNA-Seq.

We also highlighted poorly concordant genes between platforms and mapped them to biological pathways. At the gene level, only 25 of 19797 genes (0.12%) were found in all datasets as discordant, but at the pathway level, 7 of 347 pathways showed shared enrichment in discordant genes (2.02%). Most of the pathways were correlated with important technical factors, like bulk expression of TIN, so we believe that these pathways may not be accurately captured by droplet-based scRNA-seq data. However, we acknowledge that the generalizability of these pathway-level results to other biological data sets is uncertain. Additionally, when in one of the datasets the biological conditions were analyzed separately in bulk RNA-Seq and scRNA-Seq the differences in pathway enrichment results were observed. These differences were strictly related to the phenomenon under study, so again we think that this may led to misinterpretation or missing true biological knowledge.

We are aware, that the presented study has some limitations. We analyzed only 4 datasets as paired bulk and scRNA-Seq studies are extremely rare in publicly accessible repositories, especially when raw data are needed as in the presented study. However, the collected data represent broad biological experiments including cell lines and human samples from different tissues, making our findings potentially applicable to other datasets. We restricted our scRNA-Seq data to results generated by the 10x Genomics Chromium platform. We selected this platform because it provides the highest number of cells among single-cell platforms which allows averaging expressions from multiple cells, and thus can reduce random noise in the data. It is also a widely popular platform with the highest number of publicly available datasets. However, it would be valuable to analyze expression data measured using other scRNA-Seq techniques, particularly those that can investigate full-length transcripts. Since we analyzed only droplet-based data, technical factors that consider the coverage among transcripts (e.g., TIN, UTR3 length) might not be as relevant in full-length transcript-based techniques as in this study.

To summarize, in this study we revealed potential factors responsible for observed zeros and other discrepancies between platforms as well as the potential biological effects that are shifting in the scRNA-Seq. We believe that our discoveries enabled the development of new computational methods designed to correct expression shifts when comparing data from bulk and single-cell platforms. These methods could significantly improve the accuracy and reliability of single-cell transcriptomic data analysis.

## Videos

Not applicable.

## Appendix

Not applicable.

## Funding

This study was supported by the 10.13039/501100007835Silesian University of Technology grant for ground-breaking research no. 02/070/SDU/10-21-01 [JZ], the Silesian University of Technology grant for maintaining and developing research potential no. 02/070/BK_23/0043 [AP, JP], and the Silesian University of Technology rector's pro-quality grant no. 02/070/RGJ23/0042 [MM].

## Declaration of Competing Interest

C. H. is employed at HiFiBiO Therapeutics at present and was employed at Bristol-Myers Squibb in the past 36 months. L.P. has received consulting fees and honoraria from Pfizer, Astra Zeneca, Merck, Novartis, Bristol-Myers Squibb, Genentech, Eisai, Pieris, Immunomedics, Seattle Genetics, Clovis, Syndax, H3Bio, and Daiichi. All other authors don't have any competing interests.

## Data Availability

The datasets were derived from sources in the public domain: ATB263 [SRA, PRJNA657088, https://www.ncbi.nlm.nih.gov/bioproject/?term=PRJNA657088], BM [GEO, GSE120444, https://www.ncbi.nlm.nih.gov/geo/query/acc.cgi?acc=GSE120444], PR [GEO, GSE120716, https://www.ncbi.nlm.nih.gov/geo/query/acc.cgi?acc=GSE120716], and RH41 [GEO, GSE113660, https://www.ncbi.nlm.nih.gov/geo/query/acc.cgi?acc=GSE113660].
