# Supplementary material for: Evaluation of zero counts to better understand the discrepancies between bulk and single-cell RNA-Seq platforms
Source: Comput Struct Biotechnol J. 2023 Sep 29;21:4663–74. doi: 10.1016/j.csbj.2023.09.035 (PMC10568495; doi:10.1016/j.csbj.2023.09.035)
Supplement: Supplementary file 1 — Supplementary material [file mmc1.docx]

**Supplementary information for the manuscript:**

***Evaluation of zero counts to better understand the discrepancies between bulk and single-cell RNA-Seq platforms***


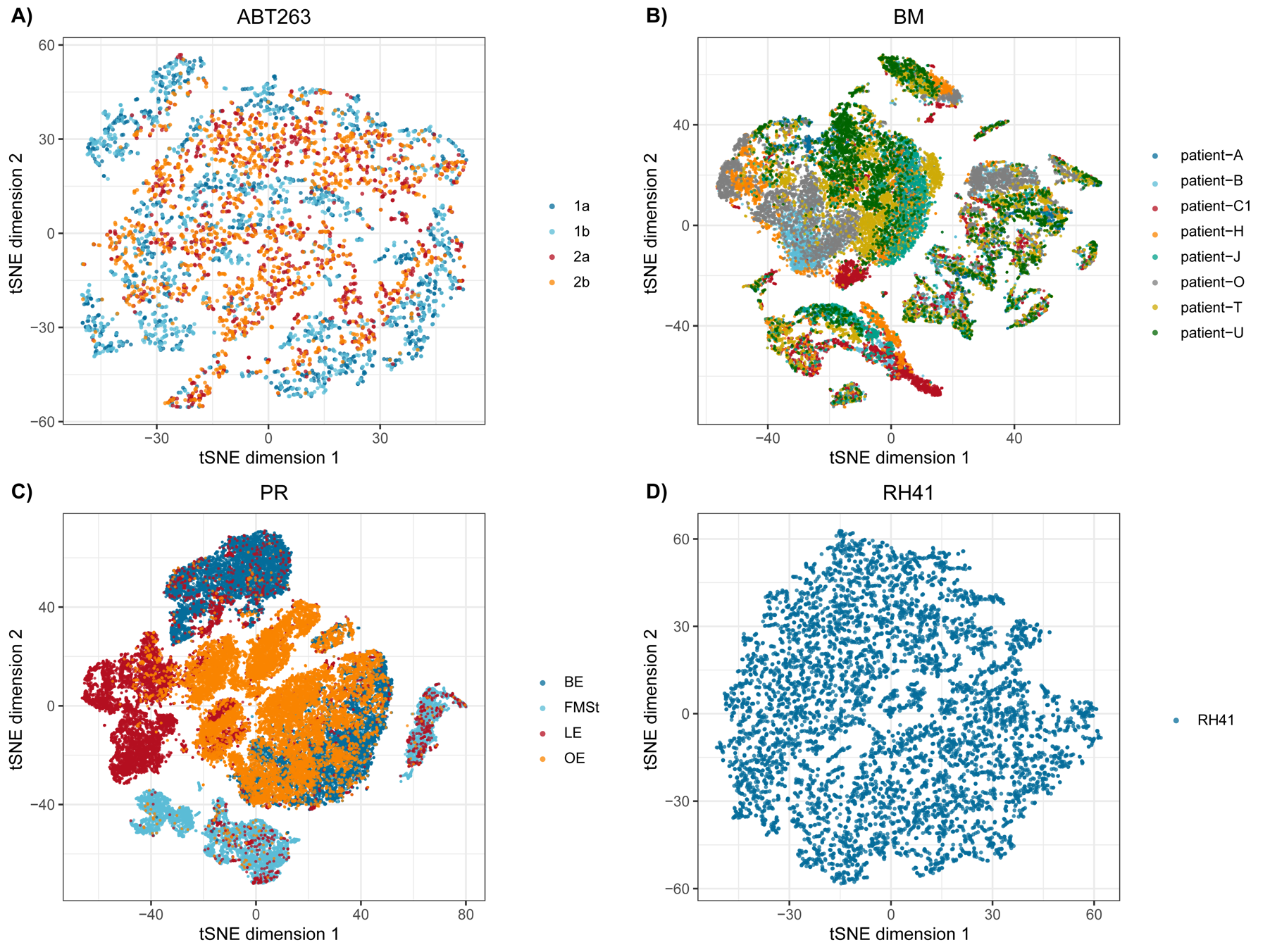


**Supplementary Figure 1. tSNE projection of scRNA-Seq platform for ABT263 (A), Bone Marrow (B), Prostate (C), and RH41 (D) datasets.** Colors identify the samples (possible batches) in each study.


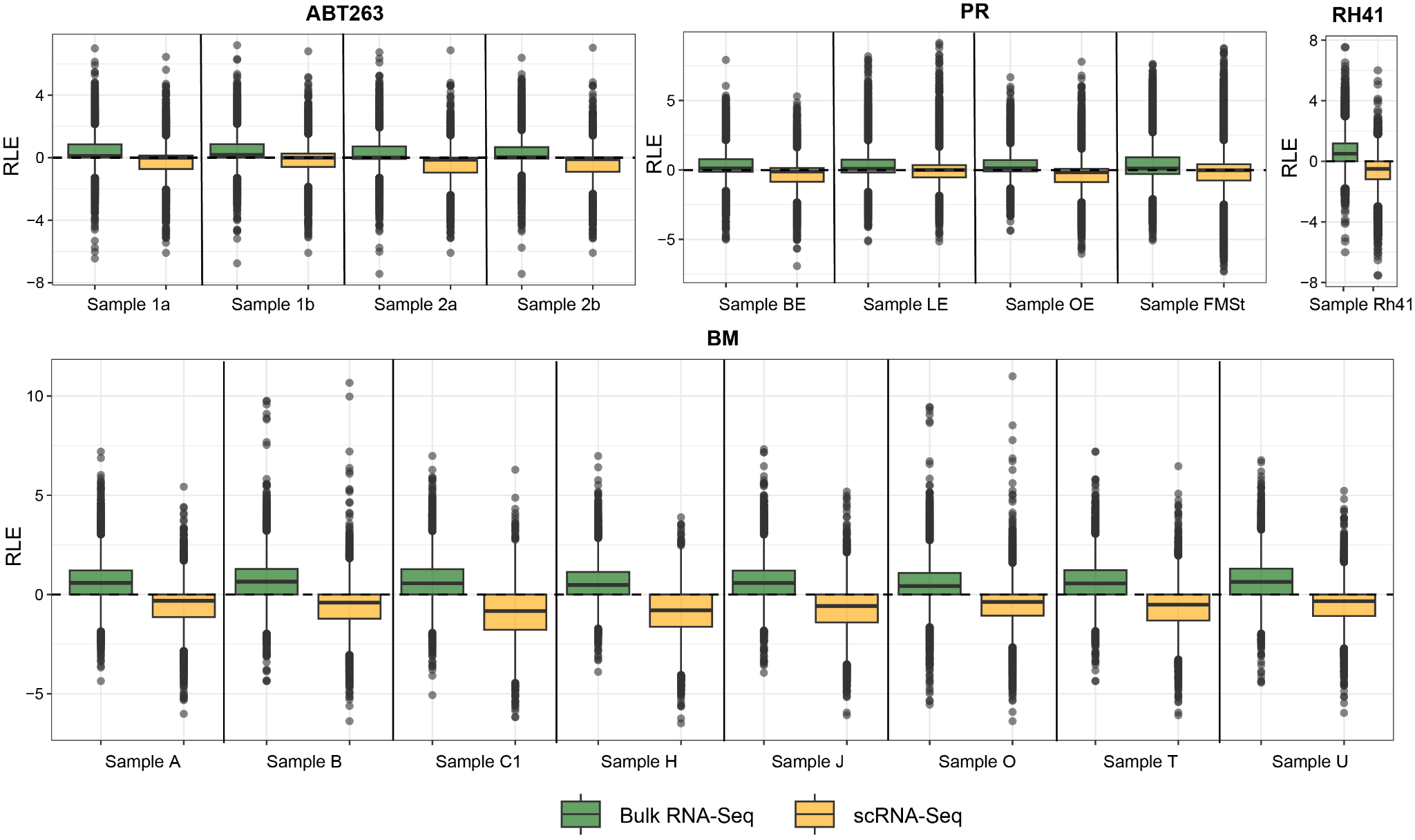


**Supplementary Figure 2. Relative log expression for ABT263, Bone Marrow (BM), Prostate (PR), and RH41 datasets.** Colors identify different platforms.


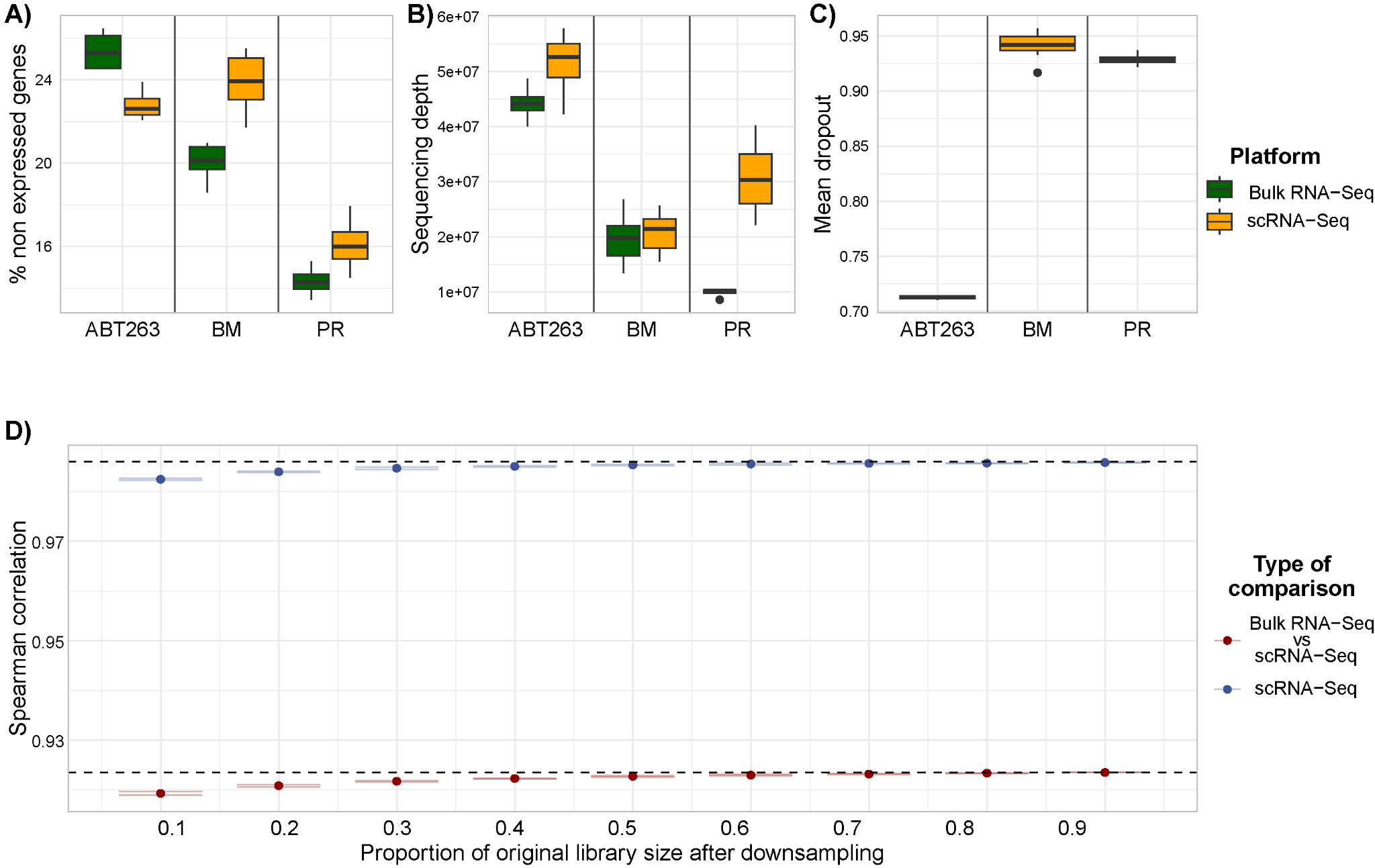


**Supplementary Figure 3. Additional characteristics of analyzed datasets and Spearman rank correlation for pairwise comparisons of different platforms of different library sizes.** A), B), and C) show the percent of non-expressed genes, sequencing depth, and mean dropout rate respectively for each sample in analyzed datasets and platforms. D) Average correlation coefficients with its 95% CI calculated between different samples grouped within the ABT263 dataset. The downsampling was performed for five random repetitions in downsampling of the original library size with different proportions.

**
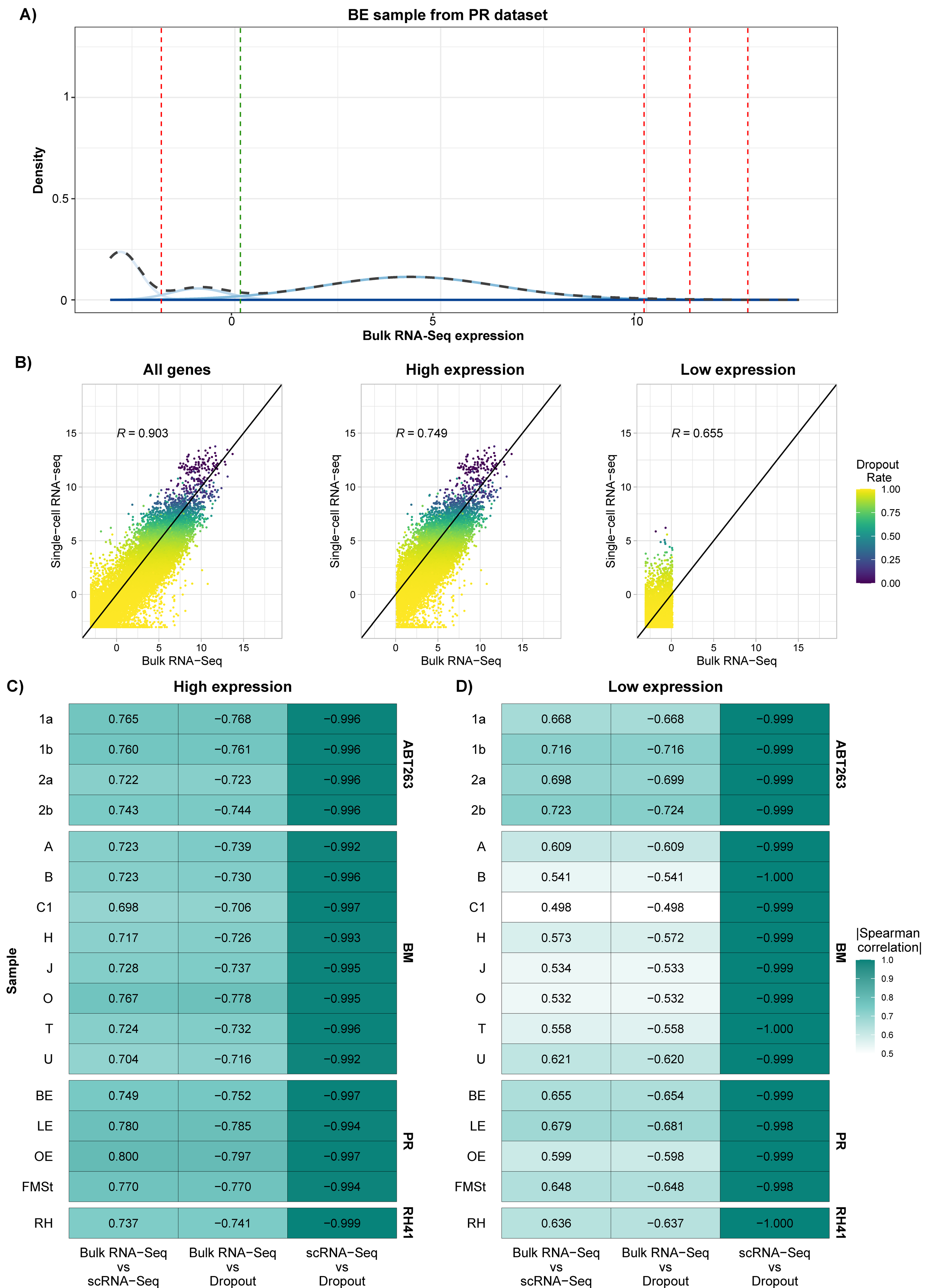
**

**Supplementary Figure 4. Pairwise correlation analysis of different platforms and dropout rate within two subgroups of genes: low and high expression.**  A) Exemplary results of finding a threshold (green vertical line) to separate genes into two groups using bulk expression data. B) Exemplary scatterplots showing discrepancies in the expression of individual genes between platforms when separated into two groups. C) and D) show Spearman rank correlation for pairwise comparisons of different platforms and dropout rates.


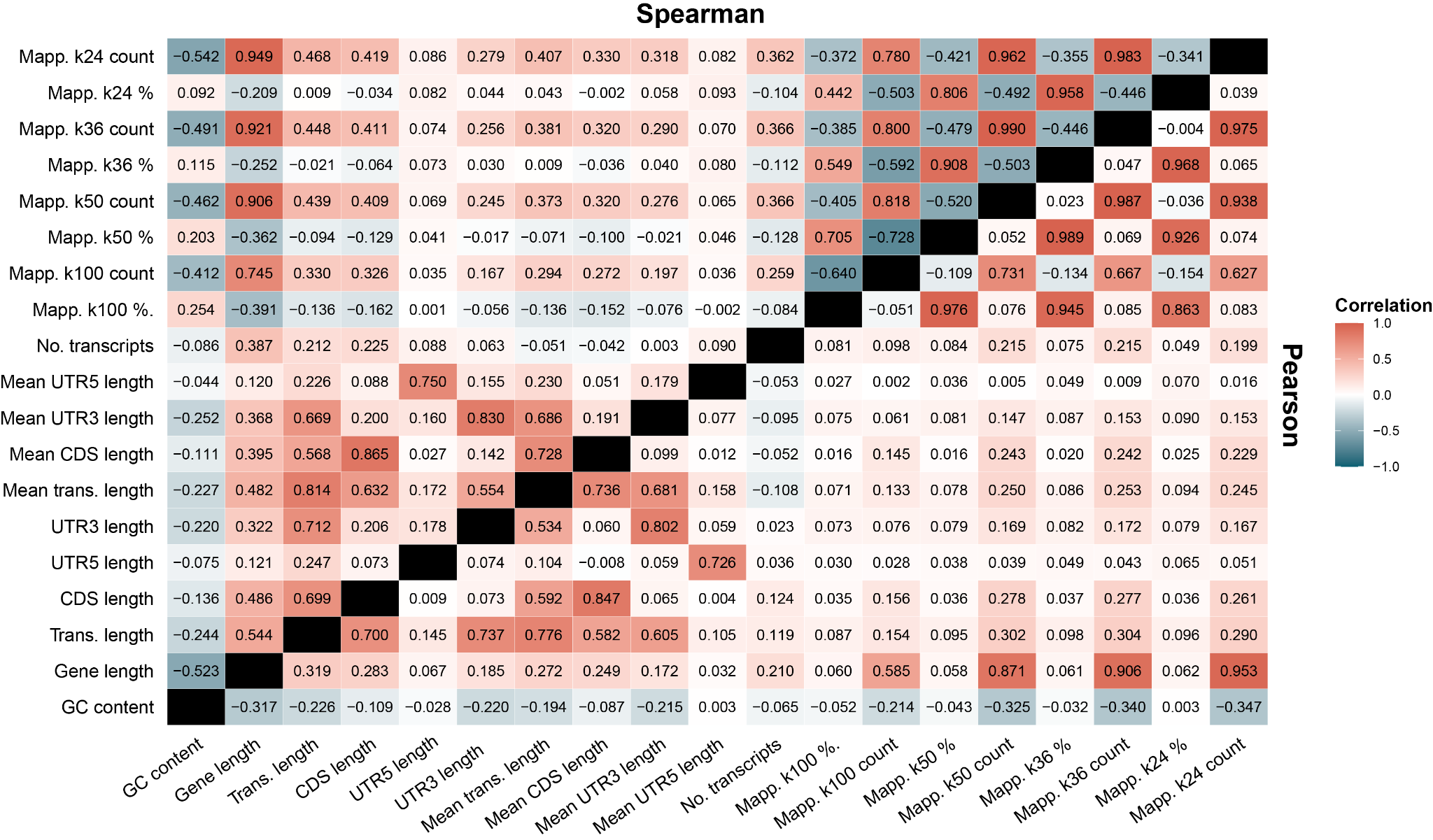


**Supplementary Figure 5. Spearman and Pearson correlations between technical factors that could potentially influence dropout rate.** Colors represent the value of the correlation coefficient. The reddish the higher the positive correlation, and the bluish the higher the negative correlation.


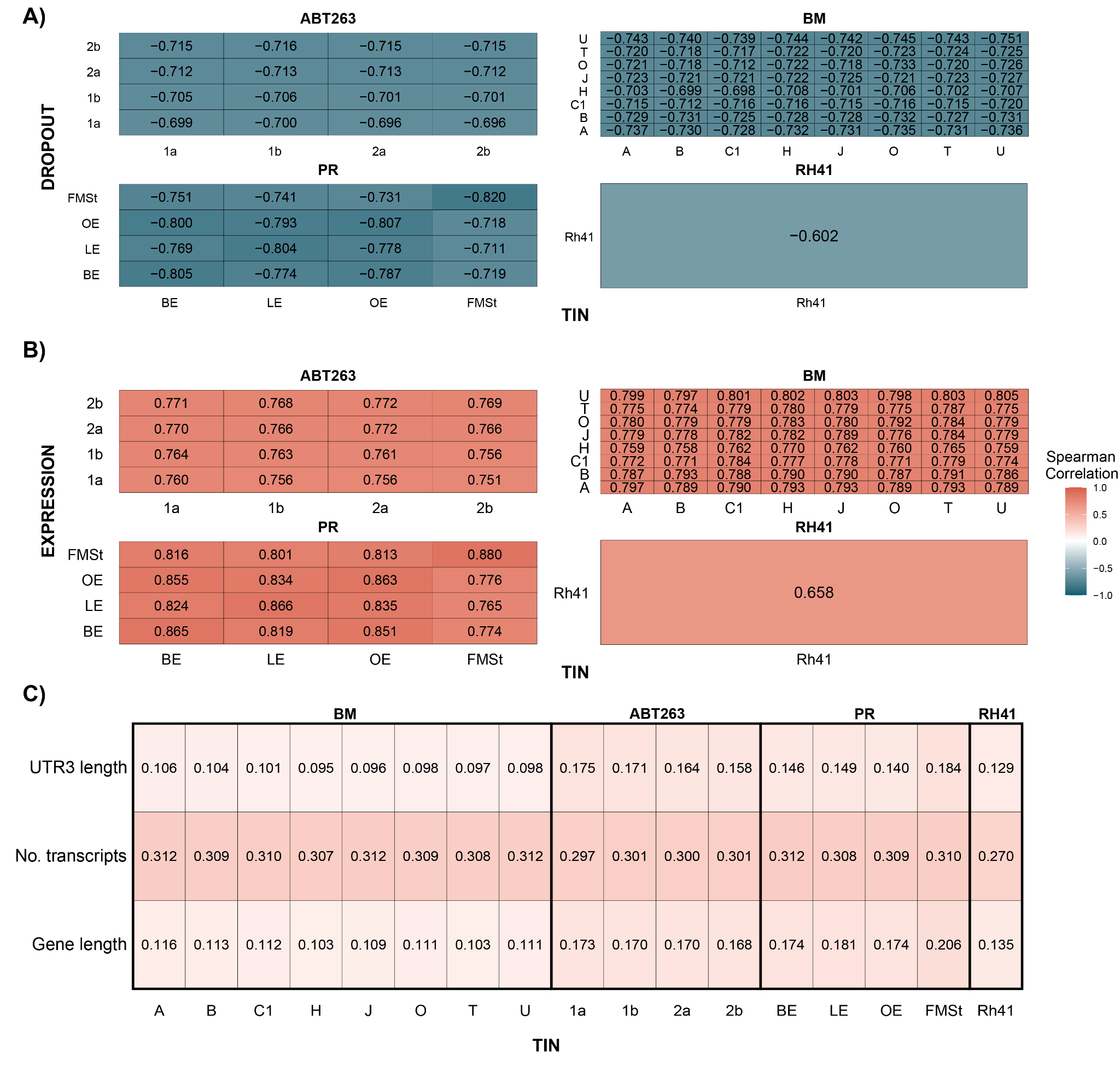


**Supplementary Figure 6. Spearman rank correlation of leading causes of dropout**. A) Correlation between TIN and dropout within each sample across the tested dataset. B) Correlation between TIN and expression within each sample across the tested dataset. C) Correlation between TIN and different transcript characteristics i.e. UTR3’ length, number of transcripts, and gene length.

**
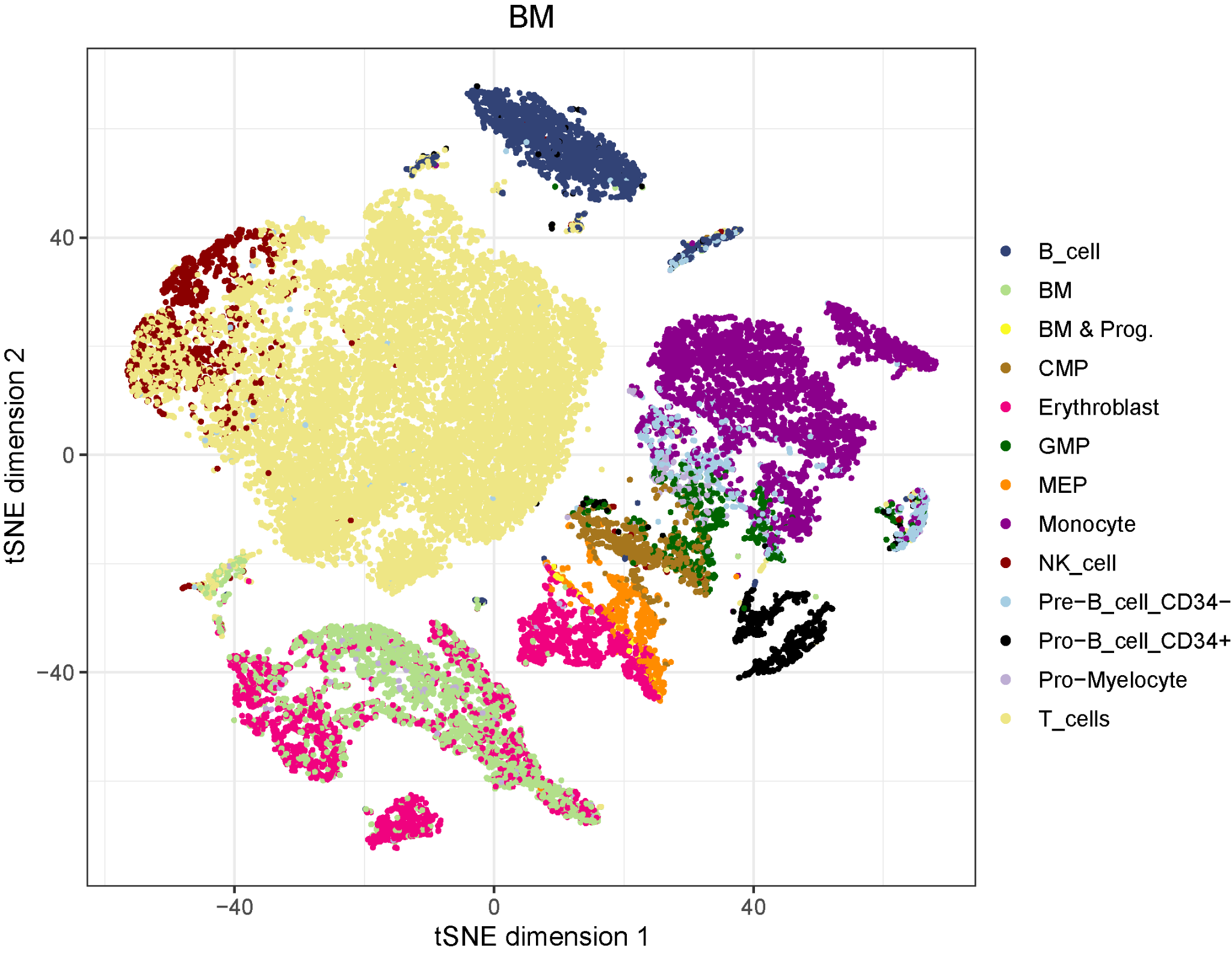
**

**Supplementary Figure 7. tSNE projection of scRNA-Seq data for BM dataset with cell types labeled using Human Primary Cell Atlas Data.**

**
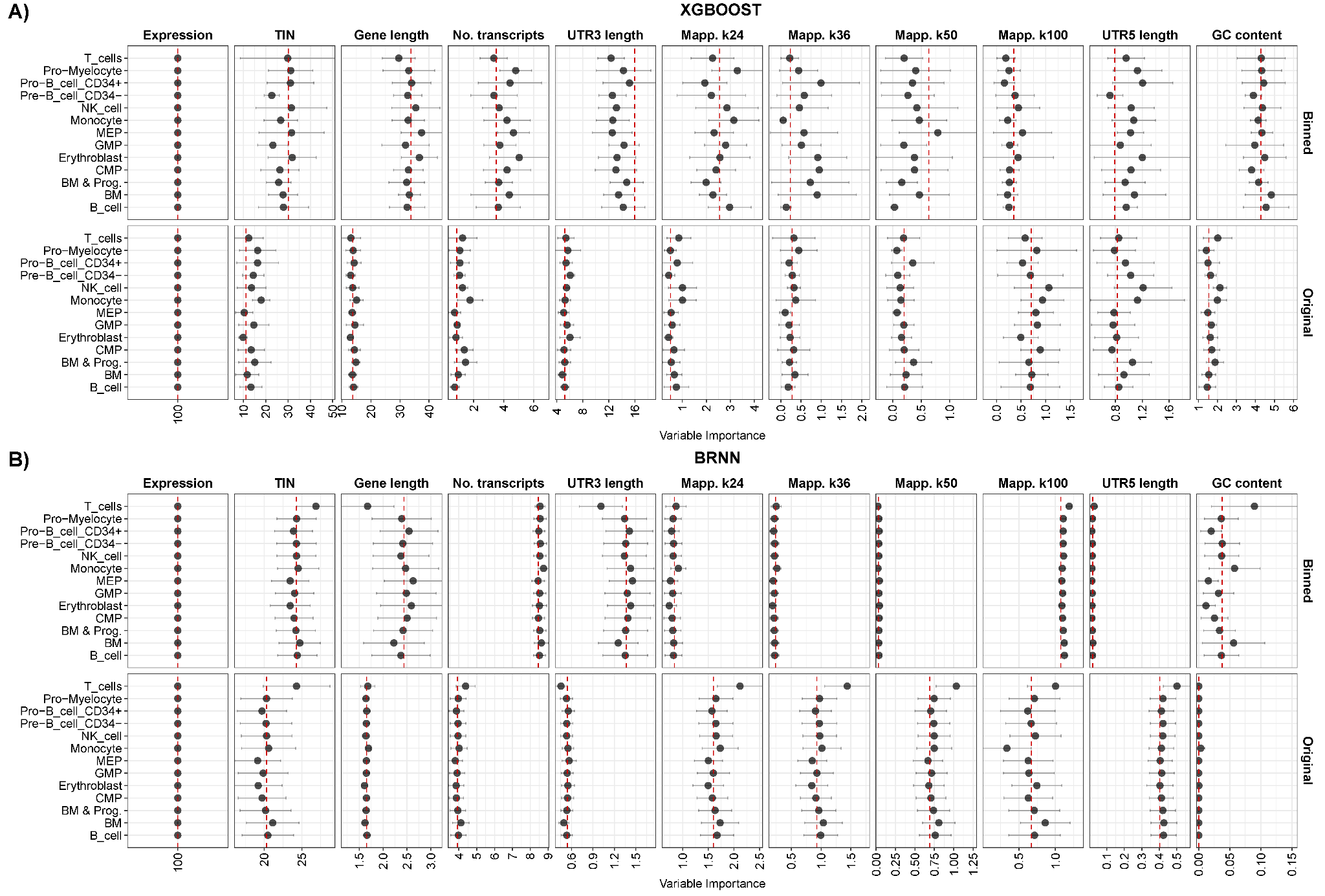
**

**Supplementary Figure 8. Variable importance from XGBOOST and BRNN models built on all factors with 95% CI for the mean (calculated from all samples within the study) regarding the impact of different cell types in the BM dataset.**


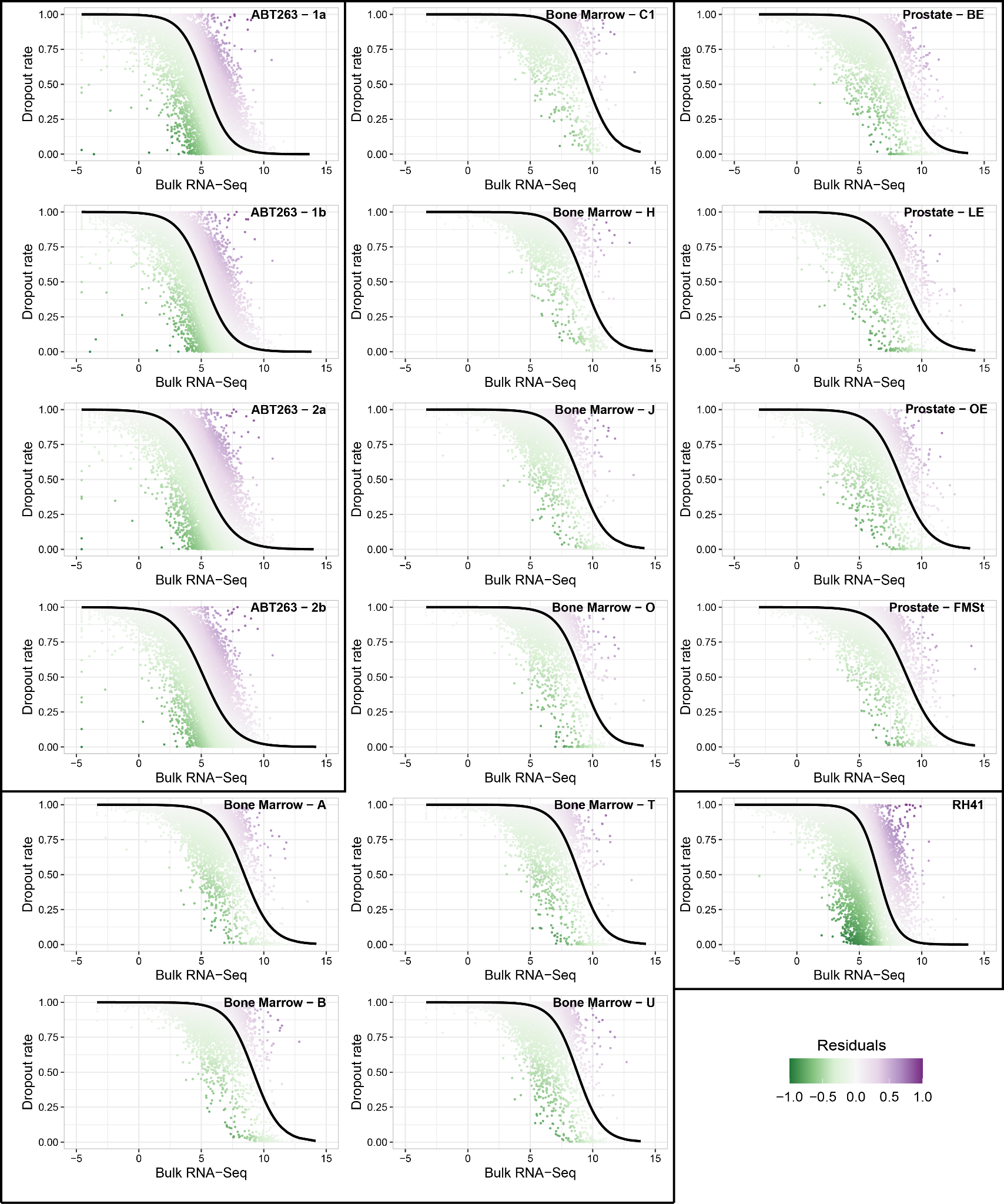


**Supplementary Figure 9. The fit of the 5PL model between Bulk RNA-Seq and dropout rate in each sample within analyzed datasets.** Colors represent the value of residuals. The more purple the higher the residual value, and the greener the higher the negative residual value. A solid black line represents a fitted model.

**
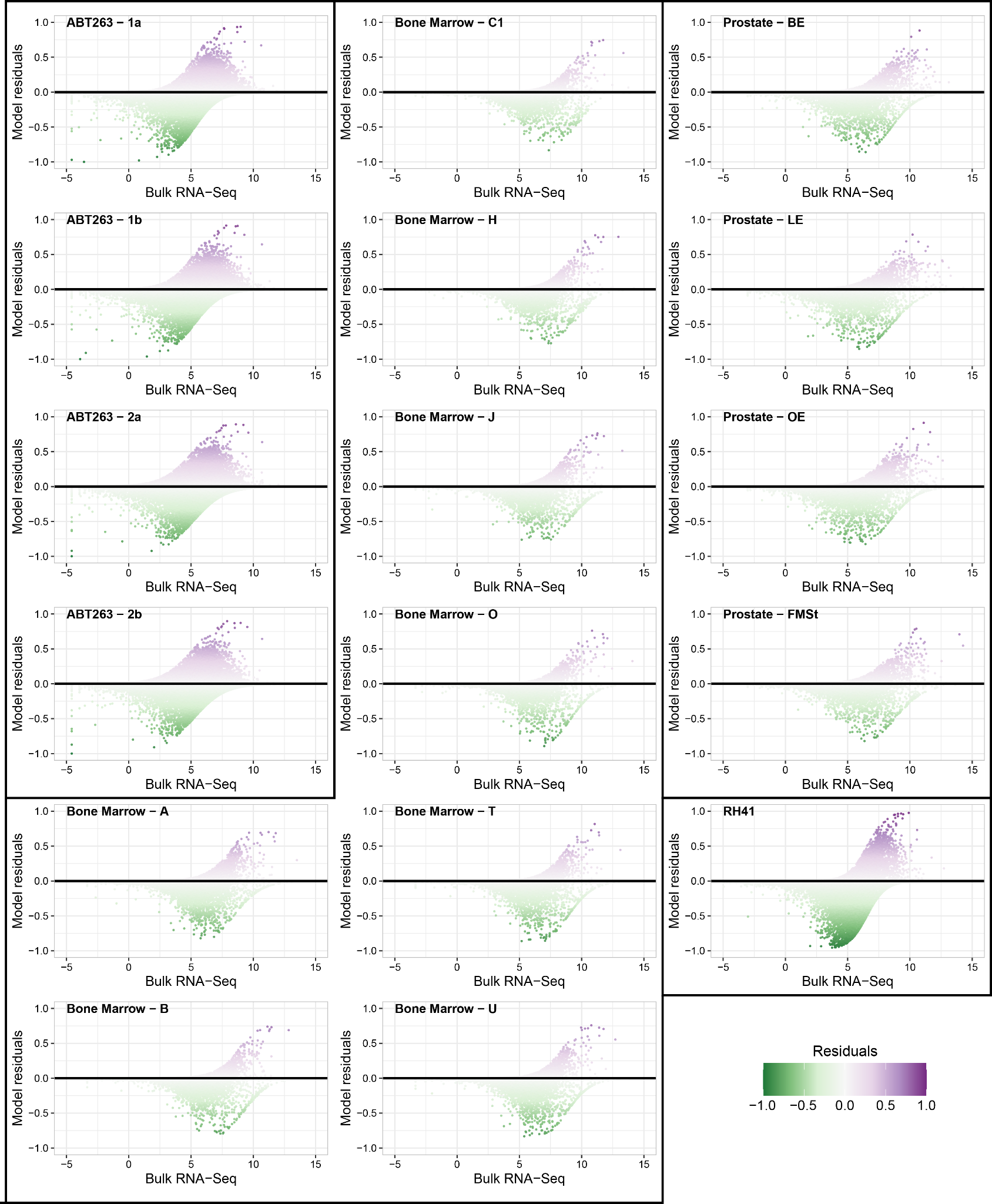
**

**Supplementary Figure 10. The residuals of the 5PL model across each sample within the analyzed dataset.** Colors represent the value of residuals. The more purple the higher the residual value, and the greener the higher the negative residual value. A solid black line is set at zero value, which represents a perfect model fit.

**
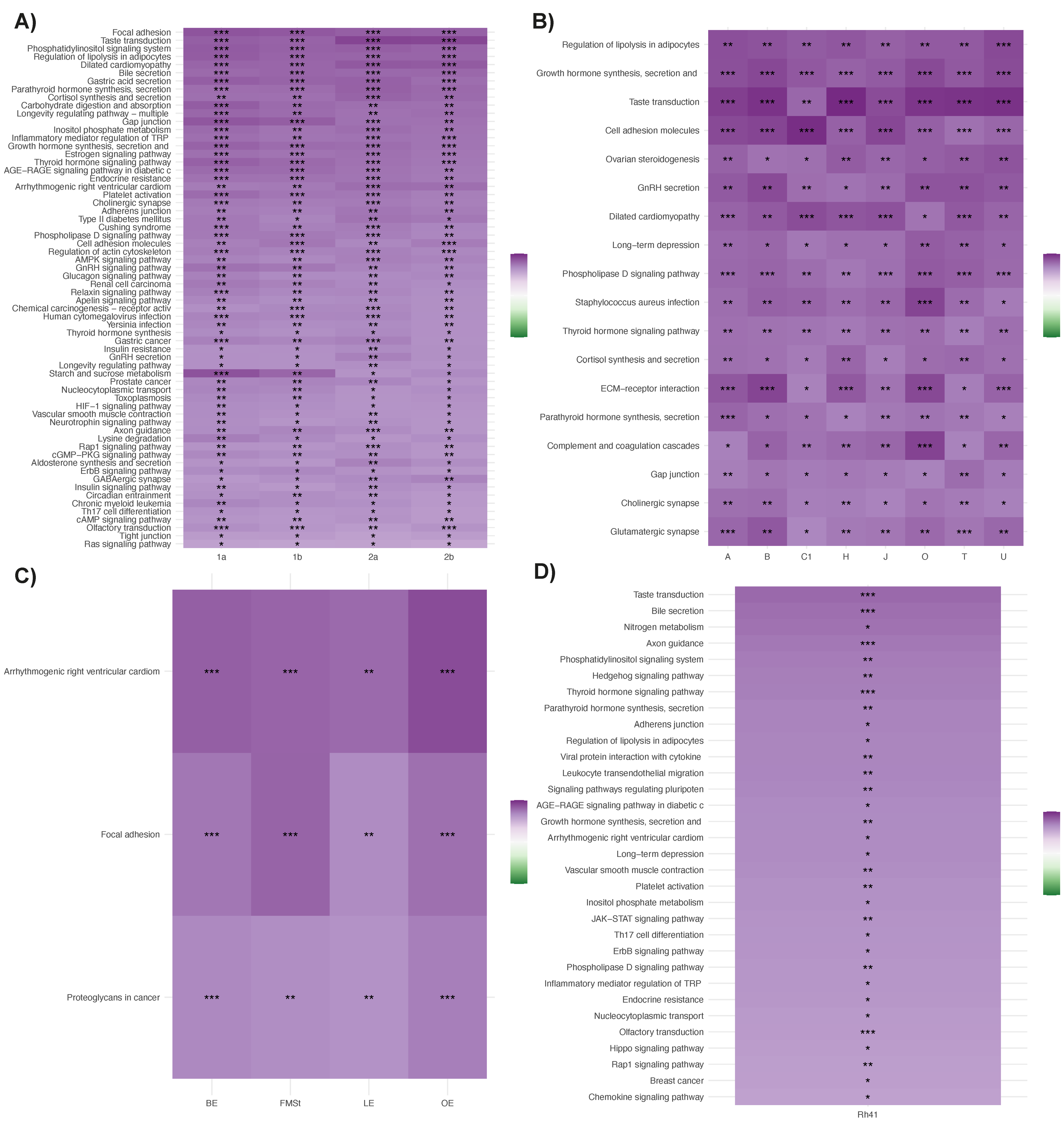
**

**Supplementary Figure 11. Heatmap of NES value from GSEA analysis of significantly up-regulated pathways between platforms for all samples within ABT263 (A), Bone Marrow (B), Prostate (C), and RH41 (D) datasets.** The intensity of the purple color represents the NES value while stars represent the test significance: * FDR < 0.05, ** FDR <0.01, ***p-value < 0.001.

**
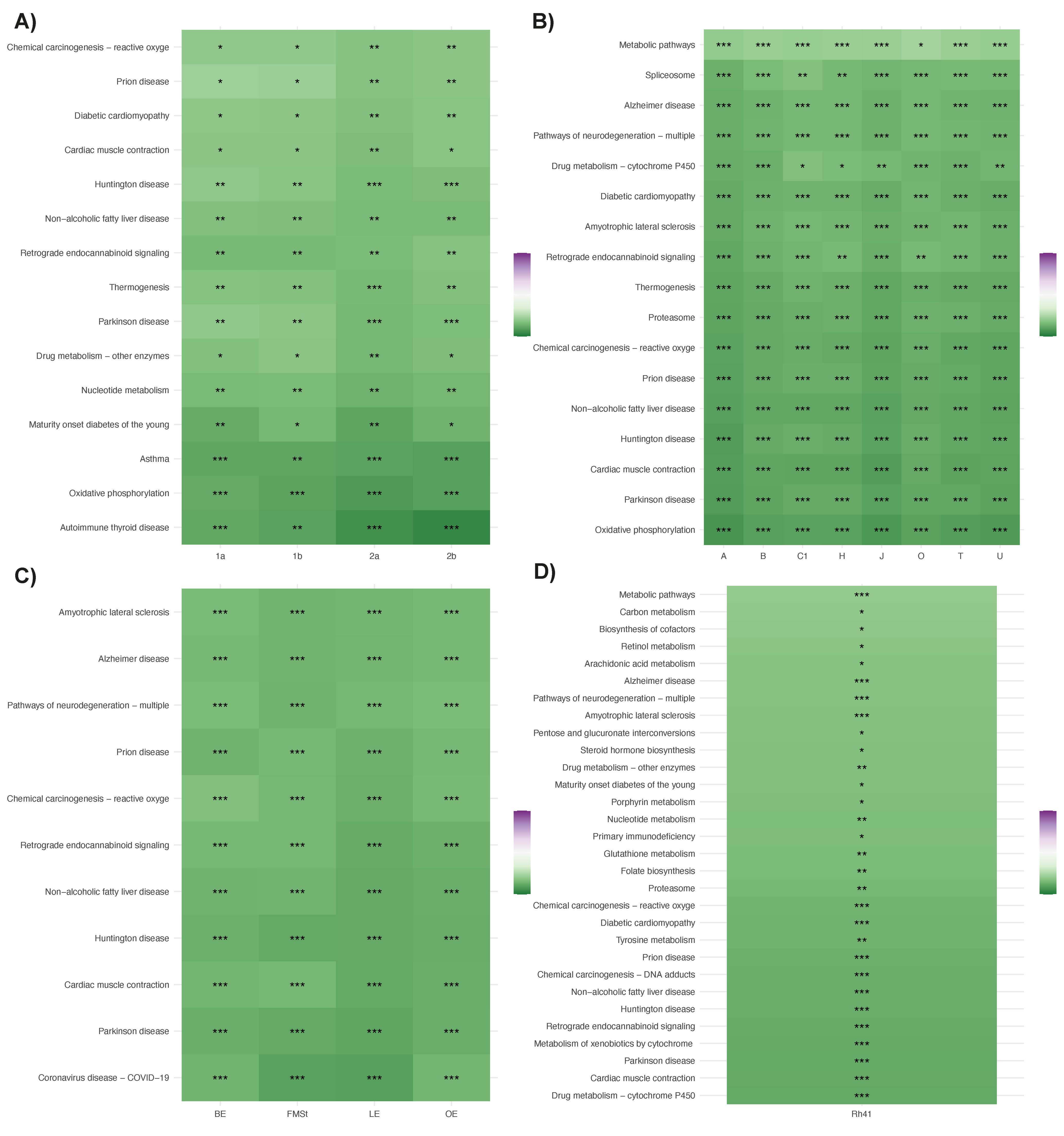
**

**Supplementary Figure 12. Heatmap of NES value from GSEA analysis of significantly down-regulated pathways between platforms for all samples within ABT263 (A), Bone Marrow (B), Prostate (C), and RH41 (D) datasets.** The intensity of the purple color represents the NES value while stars represent the test significance: * FDR < 0.05, ** FDR <0.01, ***p-value < 0.001.


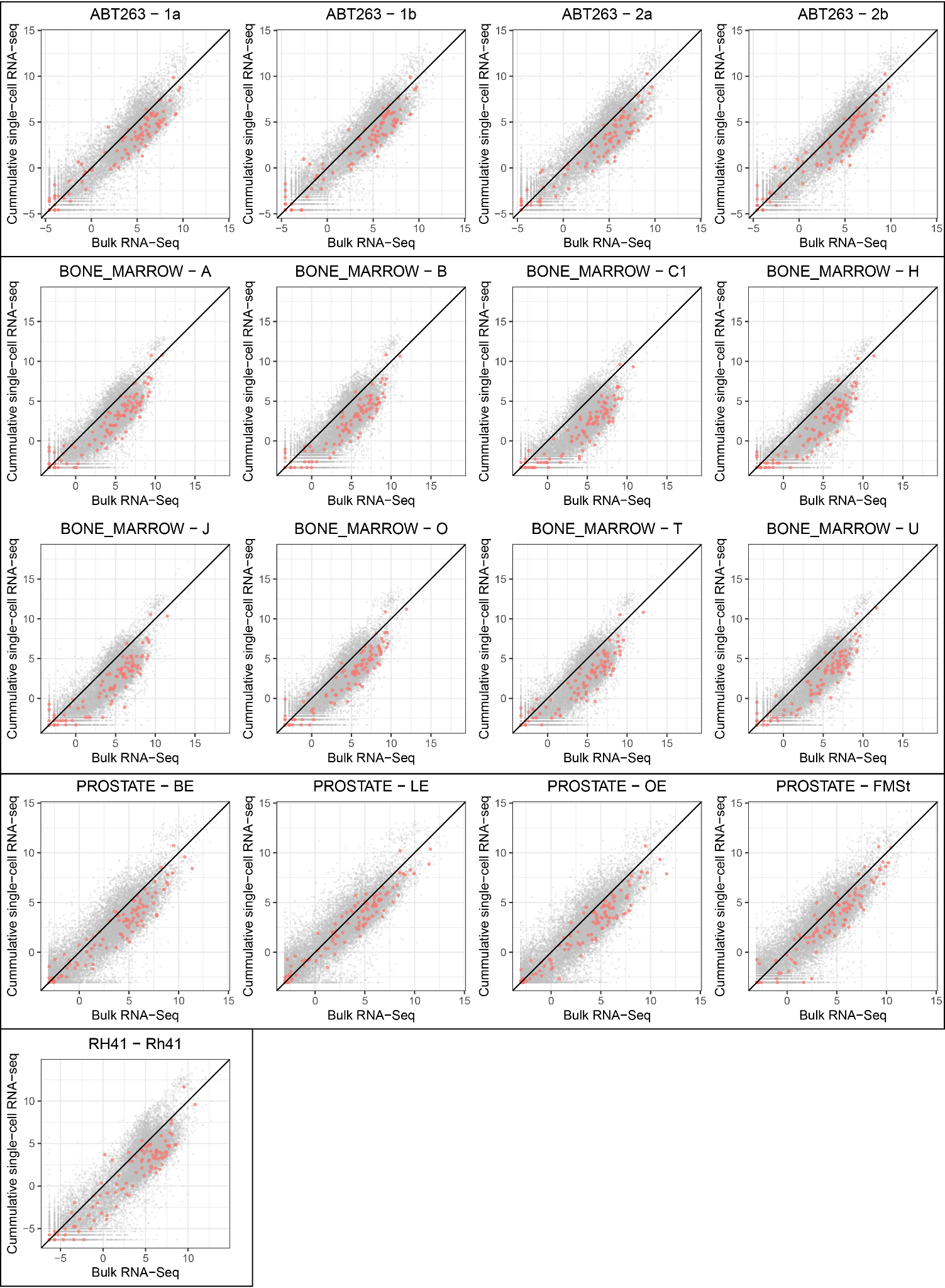


**Supplementary Figure 13. Illustration of gene expression from “Parathyroid hormone synthesis, secretion and action” KEGG pathway on Bulk RNA-Seq and summarized single-cell RNA-Seq for all analyzed samples.** Both, the x and y-axis represent normalized expression within each platform. Each dot represents one of the analyzed genes. Pink dots represent genes within the pathway of interest.

**
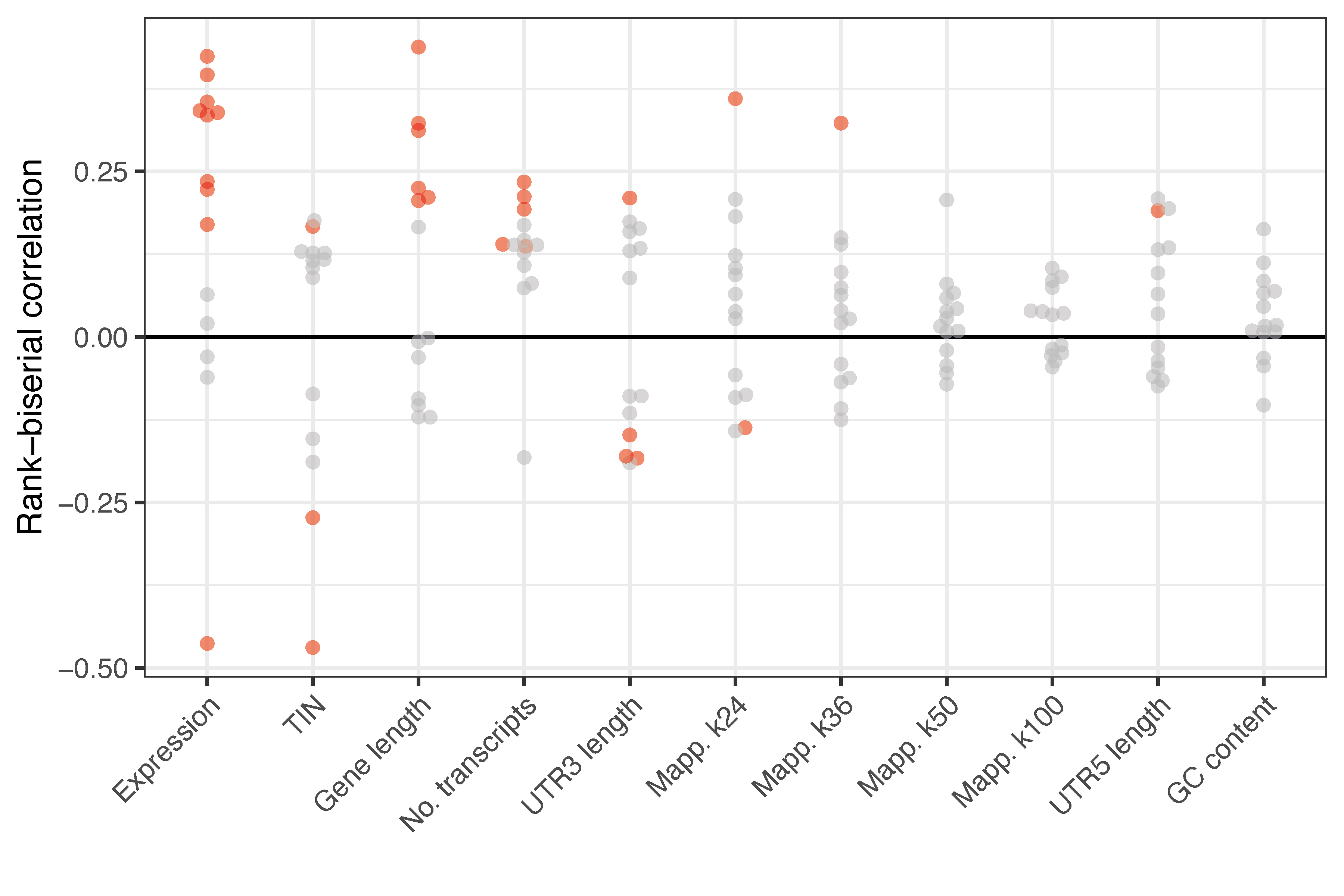
**

**Supplementary Figure 13. Rank-biserial correlation from comparing the values of technical factors of genes within each pathway versus others.** Each dot represents one pathway. Red dots represent statistically significant values (FDR < 0.05). For the “Expression” and “TIN” factors, the median value across samples is shown.

**Supplementary Table 1. A detailed number of sequenced cells in scRNA-Seq experiments for each sample and study.**

| **ABT263** | | **BM** | |
| --- | --- | --- | --- |
| **1a** | 1 232 | **A** | 2 836 |
| **1b** | 1 072 | **BM** | 3 108 |
| **2a** | 1 116 | **C1** | 3 370 |
| **2b** | 1 260 | **H** | 4 263 |
| **Total** | **4 680** | **J** | 3 257 |
| **PR** | | **O** | 4 681 |
| **BE** | 14 543 | **T** | 4 061 |
| **FMSt** | 7 498 | **U** | 3 890 |
| **LE** | 10 128 | **Total** | **29 466** |
| **OE** | 16 402 | **RH41** | |
| **Total** | **48 517** | **Rh41** | **7 004** |

**Supplementary Table 2. Results of batch effect identification by *BatchI* method.**

| **Metric** | **ABT** | **BM** | **PR** | **RH41** |
| --- | --- | --- | --- | --- |
| **test statistic** | 0.3017 | 0.9945 | 0.6344 | not applicable |
| **p-value** | 0.5989 | 0.4541 | 0.0079 |  |

**Supplementary Table 3**. **The number of zero count genes in scRNA-Seq that are expressed in Bulk RNA-Seq for each dataset and sample.**

| **ABT263** | | **BM** | |
| --- | --- | --- | --- |
| **1a** | 661 (3.34%) | **A** | 952 (4.81%) |
| **1b** | 626 (3.16%) | **B** | 1 348 (6.81%) |
| **2a** | 640 (3.23%) | **C1** | 1 742 (8.80%) |
| **2b** | 580 (2.93%) | **H** | 1 724 (8.71%) |
| **PR** | | **J** | 1 330 (6.72%) |
| **BE** | 1 002 (5.06%) | **O** | 1 412 (7.13%) |
| **FMSt** | 1 358 (6.86%) | **T** | 1 250 (6.31%) |
| **LE** | 816 (4.12%) | **U** | 1 061 (5.36%) |
| **OE** | 1 022 (5.16%) | **RH41** | |
|  | | **Rh41** | 1 139 (5.75%) |

**Supplementary Table 4. Results of ANOVA test with Tukey HSD post-hoc test for pooled correlations calculated on each analyzed platform.** Supporting results for Figure 2B.

| **Dataset** | **ANOVA p-value** | **Tukey HSD post-hoc p-value** | | | | | |
| --- | --- | --- | --- | --- | --- | --- | --- |
|  |  | **Pooled scRNA vs Pooled Bulk** | **Pooled scRNA**  **vs Pooled Dropout** | **Pooled scRNA**  **vs**  **[Bulk - scRNA]** | **Pooled Bulk**  **vs**  **Pooled Dropout** | **Pooled Bulk**  **vs**  **[Bulk - scRNA]** | **Pooled Dropout**  **vs**  **[Bulk - scRNA]** |
| **ABT** | *<0.0001* | 0.9987 | ~1 | *<0.0001* | 0.9987 | *<0.0001* | *<0.0001* |
| **BM** | *<0.0001* | *0.0111* | 0.9987 | *<0.0001* | 0.0176 | *<0.0001* | *<0.0001* |
| **PR** | 0.5731 | 0.7816 | 0.9999 | 0.9921 | 0.7980 | 0.4972 | 0.9886 |

**Supplementary Table 5. The number of genes with values of residuals higher than a threshold.**

| **Dataset** | **Sample** | **Range of scaled residuals** | | | |
| --- | --- | --- | --- | --- | --- |
|  |  | **<-1;1>** | **<-2;-1> or <1;2>** | **<-3;-2> or <2;3>** | **<-3 or >3** |
| **ABT** | 1a | 15 255 | 2 984 | 1 192 | 366 |
|  | 1b | 15 227 | 3 042 | 1 180 | 348 |
|  | 2a | 15 021 | 3 214 | 1 271 | 291 |
|  | 2b | 15 078 | 3 140 | 1 267 | 312 |
| **BM** | A | 16 906 | 1 676 | 652 | 563 |
|  | B | 17 396 | 1 370 | 503 | 528 |
|  | C1 | 17 617 | 1 207 | 447 | 526 |
|  | H | 17 658 | 1 167 | 442 | 530 |
|  | J | 17 310 | 1 402 | 537 | 548 |
|  | O | 17 707 | 1 114 | 446 | 530 |
|  | T | 17 412 | 1 336 | 508 | 541 |
|  | U | 17 282 | 1 389 | 572 | 554 |
| **PR** | BE | 17 297 | 1 322 | 593 | 585 |
|  | FMSt | 17 423 | 1 302 | 517 | 555 |
|  | LE | 17 428 | 1 287 | 521 | 561 |
|  | OE | 17 229 | 1 391 | 589 | 588 |
| **RH41** | RH41 | 15 772 | 2 420 | 1 117 | 488 |

**Supplementary Table 6. ML models fit metrics.**

| **Dataset** | **Sample** | **Model** | **Feature type** | **R^2^** | **RMSE** | **MAE** |
| --- | --- | --- | --- | --- | --- | --- |
| ABT263 | 1a | BRNN | Binned | 0.787 | 0.155 | 0.099 |
| ABT263 | 1a | BRNN | Original | 0.838 | 0.135 | 0.084 |
| ABT263 | 1a | XGBOOST | Binned | 0.793 | 0.153 | 0.098 |
| ABT263 | 1a | XGBOOST | Original | 0.852 | 0.129 | 0.081 |
| ABT263 | 1b | BRNN | Binned | 0.798 | 0.151 | 0.096 |
| ABT263 | 1b | BRNN | Original | 0.834 | 0.136 | 0.086 |
| ABT263 | 1b | XGBOOST | Binned | 0.804 | 0.149 | 0.095 |
| ABT263 | 1b | XGBOOST | Original | 0.869 | 0.121 | 0.076 |
| ABT263 | 2a | BRNN | Binned | 0.791 | 0.158 | 0.099 |
| ABT263 | 2a | BRNN | Original | 0.829 | 0.143 | 0.087 |
| ABT263 | 2a | XGBOOST | Binned | 0.794 | 0.157 | 0.099 |
| ABT263 | 2a | XGBOOST | Original | 0.849 | 0.134 | 0.084 |
| ABT263 | 2b | BRNN | Binned | 0.796 | 0.155 | 0.097 |
| ABT263 | 2b | BRNN | Original | 0.836 | 0.139 | 0.085 |
| ABT263 | 2b | XGBOOST | Binned | 0.802 | 0.152 | 0.096 |
| ABT263 | 2b | XGBOOST | Original | 0.865 | 0.126 | 0.078 |
| BM | A | BRNN | Binned | 0.589 | 0.096 | 0.049 |
| BM | A | BRNN | Original | 0.728 | 0.078 | 0.042 |
| BM | A | XGBOOST | Binned | 0.621 | 0.092 | 0.048 |
| BM | A | XGBOOST | Original | 0.783 | 0.070 | 0.039 |
| BM | B | BRNN | Binned | 0.539 | 0.081 | 0.037 |
| BM | B | BRNN | Original | 0.714 | 0.064 | 0.030 |
| BM | B | XGBOOST | Binned | 0.582 | 0.077 | 0.036 |
| BM | B | XGBOOST | Original | 0.792 | 0.054 | 0.028 |
| BM | C1 | BRNN | Binned | 0.494 | 0.070 | 0.032 |
| BM | C1 | BRNN | Original | 0.668 | 0.057 | 0.026 |
| BM | C1 | XGBOOST | Binned | 0.557 | 0.066 | 0.030 |
| BM | C1 | XGBOOST | Original | 0.772 | 0.047 | 0.024 |
| BM | H | BRNN | Binned | 0.509 | 0.076 | 0.033 |
| BM | H | BRNN | Original | 0.700 | 0.060 | 0.028 |
| BM | H | XGBOOST | Binned | 0.565 | 0.072 | 0.032 |
| BM | H | XGBOOST | Original | 0.802 | 0.049 | 0.024 |
| BM | J | BRNN | Binned | 0.545 | 0.083 | 0.039 |
| BM | J | BRNN | Original | 0.686 | 0.069 | 0.035 |
| BM | J | XGBOOST | Binned | 0.580 | 0.080 | 0.038 |
| BM | J | XGBOOST | Original | 0.789 | 0.057 | 0.030 |
| BM | O | BRNN | Binned | 0.525 | 0.084 | 0.036 |
| BM | O | BRNN | Original | 0.743 | 0.062 | 0.027 |
| BM | O | XGBOOST | Binned | 0.583 | 0.078 | 0.035 |
| BM | O | XGBOOST | Original | 0.809 | 0.053 | 0.026 |
| BM | T | BRNN | Binned | 0.544 | 0.088 | 0.041 |
| BM | T | BRNN | Original | 0.716 | 0.069 | 0.034 |
| BM | T | XGBOOST | Binned | 0.587 | 0.084 | 0.040 |
| BM | T | XGBOOST | Original | 0.803 | 0.058 | 0.031 |
| BM | U | BRNN | Binned | 0.544 | 0.092 | 0.044 |
| BM | U | BRNN | Original | 0.686 | 0.077 | 0.038 |
| BM | U | XGBOOST | Binned | 0.588 | 0.088 | 0.042 |
| BM | U | XGBOOST | Original | 0.778 | 0.065 | 0.034 |
| PR | BE | BRNN | Binned | 0.542 | 0.101 | 0.047 |
| PR | BE | BRNN | Original | 0.719 | 0.079 | 0.039 |
| PR | BE | XGBOOST | Binned | 0.559 | 0.099 | 0.048 |
| PR | BE | XGBOOST | Original | 0.756 | 0.074 | 0.037 |
| PR | FMSt | BRNN | Binned | 0.533 | 0.092 | 0.042 |
| PR | FMSt | BRNN | Original | 0.727 | 0.070 | 0.033 |
| PR | FMSt | XGBOOST | Binned | 0.556 | 0.090 | 0.041 |
| PR | FMSt | XGBOOST | Original | 0.772 | 0.064 | 0.031 |
| PR | LE | BRNN | Binned | 0.557 | 0.094 | 0.044 |
| PR | LE | BRNN | Original | 0.720 | 0.075 | 0.036 |
| PR | LE | XGBOOST | Binned | 0.580 | 0.092 | 0.044 |
| PR | LE | XGBOOST | Original | 0.762 | 0.069 | 0.035 |
| PR | OE | BRNN | Binned | 0.545 | 0.105 | 0.051 |
| PR | OE | BRNN | Original | 0.706 | 0.085 | 0.042 |
| PR | OE | XGBOOST | Binned | 0.569 | 0.103 | 0.050 |
| PR | OE | XGBOOST | Original | 0.743 | 0.079 | 0.041 |
| RH41 | Rh41 | BRNN | Binned | 0.666 | 0.163 | 0.101 |
| RH41 | Rh41 | BRNN | Original | 0.720 | 0.149 | 0.092 |
| RH41 | Rh41 | XGBOOST | Binned | 0.689 | 0.157 | 0.098 |
| RH41 | Rh41 | XGBOOST | Original | 0.785 | 0.130 | 0.082 |

**Supplementary Table 7. Statistical analysis comparing the values of technical factors of genes within each pathway versus others.**

| **Technical Factor** | **Rank-biserial correlation** | **P-value** | **FDR** | **Pathway** |
| --- | --- | --- | --- | --- |
| Expression | 0.355 | 8.37E-18 | 1.26E-15 | Chemical carcinogenesis - reactive oxygen species |
| Expression | 0.235 | 8.58E-06 | 1.18E-03 | Growth hormone synthesis, secretion and action |
| Expression | 0.339 | 1.79E-22 | 2.74E-20 | Huntington disease |
| Expression | 0.424 | 1.71E-19 | 2.59E-17 | Non-alcoholic fatty liver disease |
| Expression | 0.223 | 6.33E-05 | 8.23E-03 | Parathyroid hormone synthesis, secretion and action |
| Expression | 0.396 | 4.82E-26 | 7.42E-24 | Parkinson disease |
| Expression | 0.17 | 3.68E-04 | 4.57E-02 | Phospholipase D signaling pathway |
| Expression | 0.335 | 1.16E-19 | 1.76E-17 | Prion disease |
| Expression | -0.463 | 1.33E-13 | 1.97E-11 | Taste transduction |
| Expression | 0.342 | 6.68E-11 | 9.69E-09 | Thyroid hormone signaling pathway |
| Expression | -0.0609 | 3.31E-01 | 1.00E+00 | Arrhythmogenic right ventricular cardiomyopathy |
| Expression | -0.03 | 6.33E-01 | 1.00E+00 | Cardiac muscle contraction |
| Expression | 0.0641 | 4.14E-01 | 1.00E+00 | Regulation of lipolysis in adipocytes |
| Expression | 0.0205 | 6.73E-01 | 1.00E+00 | Retrograde endocannabinoid signaling |
| GC content | -0.0319 | 6.29E-01 | 1.00E+00 | Arrhythmogenic right ventricular cardiomyopathy |
| GC content | 0.163 | 1.05E-02 | 9.73E-01 | Cardiac muscle contraction |
| GC content | 0.00788 | 8.49E-01 | 1.00E+00 | Chemical carcinogenesis - reactive oxygen species |
| GC content | 0.0846 | 1.11E-01 | 1.00E+00 | Growth hormone synthesis, secretion and action |
| GC content | 0.018 | 6.05E-01 | 1.00E+00 | Huntington disease |
| GC content | 0.0663 | 1.60E-01 | 1.00E+00 | Non-alcoholic fatty liver disease |
| GC content | 0.112 | 4.59E-02 | 1.00E+00 | Parathyroid hormone synthesis, secretion and action |
| GC content | 0.0691 | 6.63E-02 | 1.00E+00 | Parkinson disease |
| GC content | 0.00749 | 8.75E-01 | 1.00E+00 | Phospholipase D signaling pathway |
| GC content | 0.046 | 2.14E-01 | 1.00E+00 | Prion disease |
| GC content | 0.00938 | 9.05E-01 | 1.00E+00 | Regulation of lipolysis in adipocytes |
| GC content | -0.044 | 3.67E-01 | 1.00E+00 | Retrograde endocannabinoid signaling |
| GC content | -0.103 | 1.01E-01 | 1.00E+00 | Taste transduction |
| GC content | 0.0165 | 7.54E-01 | 1.00E+00 | Thyroid hormone signaling pathway |
| Gene length | 0.438 | 3.06E-11 | 4.47E-09 | Arrhythmogenic right ventricular cardiomyopathy |
| Gene length | 0.225 | 2.26E-05 | 3.01E-03 | Growth hormone synthesis, secretion and action |
| Gene length | 0.211 | 1.79E-04 | 2.29E-02 | Parathyroid hormone synthesis, secretion and action |
| Gene length | 0.323 | 1.47E-11 | 2.16E-09 | Phospholipase D signaling pathway |
| Gene length | 0.206 | 2.45E-05 | 3.23E-03 | Retrograde endocannabinoid signaling |
| Gene length | 0.312 | 3.14E-09 | 4.52E-07 | Thyroid hormone signaling pathway |
| Gene length | -0.00159 | 9.80E-01 | 1.00E+00 | Cardiac muscle contraction |
| Gene length | -0.0307 | 4.59E-01 | 1.00E+00 | Chemical carcinogenesis - reactive oxygen species |
| Gene length | -0.00675 | 8.46E-01 | 1.00E+00 | Huntington disease |
| Gene length | -0.121 | 1.05E-02 | 9.73E-01 | Non-alcoholic fatty liver disease |
| Gene length | -0.121 | 1.31E-03 | 1.47E-01 | Parkinson disease |
| Gene length | -0.0931 | 1.19E-02 | 1.00E+00 | Prion disease |
| Gene length | 0.166 | 3.46E-02 | 1.00E+00 | Regulation of lipolysis in adipocytes |
| Gene length | -0.103 | 1.02E-01 | 1.00E+00 | Taste transduction |
| Mapp. k100 | -0.0453 | 4.16E-01 | 1.00E+00 | Arrhythmogenic right ventricular cardiomyopathy |
| Mapp. k100 | 0.0909 | 8.99E-02 | 1.00E+00 | Cardiac muscle contraction |
| Mapp. k100 | -0.0279 | 4.24E-01 | 1.00E+00 | Chemical carcinogenesis - reactive oxygen species |
| Mapp. k100 | -0.024 | 5.92E-01 | 1.00E+00 | Growth hormone synthesis, secretion and action |
| Mapp. k100 | 0.0358 | 2.23E-01 | 1.00E+00 | Huntington disease |
| Mapp. k100 | 0.104 | 8.78E-03 | 8.44E-01 | Non-alcoholic fatty liver disease |
| Mapp. k100 | 0.0396 | 4.04E-01 | 1.00E+00 | Parathyroid hormone synthesis, secretion and action |
| Mapp. k100 | 0.0852 | 7.35E-03 | 7.43E-01 | Parkinson disease |
| Mapp. k100 | -0.0124 | 7.58E-01 | 1.00E+00 | Phospholipase D signaling pathway |
| Mapp. k100 | 0.0336 | 2.83E-01 | 1.00E+00 | Prion disease |
| Mapp. k100 | 0.0384 | 5.63E-01 | 1.00E+00 | Regulation of lipolysis in adipocytes |
| Mapp. k100 | -0.0182 | 6.59E-01 | 1.00E+00 | Retrograde endocannabinoid signaling |
| Mapp. k100 | 0.0748 | 1.58E-01 | 1.00E+00 | Taste transduction |
| Mapp. k100 | -0.0365 | 4.11E-01 | 1.00E+00 | Thyroid hormone signaling pathway |
| Mapp. k24 | -0.137 | 7.87E-05 | 1.02E-02 | Huntington disease |
| Mapp. k24 | 0.36 | 9.59E-09 | 1.37E-06 | Taste transduction |
| Mapp. k24 | 0.0649 | 3.25E-01 | 1.00E+00 | Arrhythmogenic right ventricular cardiomyopathy |
| Mapp. k24 | 0.0385 | 5.45E-01 | 1.00E+00 | Cardiac muscle contraction |
| Mapp. k24 | -0.142 | 6.06E-04 | 7.22E-02 | Chemical carcinogenesis - reactive oxygen species |
| Mapp. k24 | 0.0937 | 7.75E-02 | 1.00E+00 | Growth hormone synthesis, secretion and action |
| Mapp. k24 | -0.0575 | 2.23E-01 | 1.00E+00 | Non-alcoholic fatty liver disease |
| Mapp. k24 | 0.182 | 1.23E-03 | 1.40E-01 | Parathyroid hormone synthesis, secretion and action |
| Mapp. k24 | -0.0913 | 1.53E-02 | 1.00E+00 | Parkinson disease |
| Mapp. k24 | 0.123 | 9.83E-03 | 9.24E-01 | Phospholipase D signaling pathway |
| Mapp. k24 | -0.0873 | 1.84E-02 | 1.00E+00 | Prion disease |
| Mapp. k24 | 0.208 | 8.29E-03 | 8.13E-01 | Regulation of lipolysis in adipocytes |
| Mapp. k24 | 0.104 | 3.28E-02 | 1.00E+00 | Retrograde endocannabinoid signaling |
| Mapp. k24 | 0.0277 | 5.99E-01 | 1.00E+00 | Thyroid hormone signaling pathway |
| Mapp. k36 | 0.323 | 2.52E-07 | 3.57E-05 | Taste transduction |
| Mapp. k36 | 0.0212 | 7.47E-01 | 1.00E+00 | Arrhythmogenic right ventricular cardiomyopathy |
| Mapp. k36 | 0.04 | 5.29E-01 | 1.00E+00 | Cardiac muscle contraction |
| Mapp. k36 | -0.125 | 2.62E-03 | 2.80E-01 | Chemical carcinogenesis - reactive oxygen species |
| Mapp. k36 | 0.063 | 2.35E-01 | 1.00E+00 | Growth hormone synthesis, secretion and action |
| Mapp. k36 | -0.108 | 1.84E-03 | 2.04E-01 | Huntington disease |
| Mapp. k36 | -0.041 | 3.85E-01 | 1.00E+00 | Non-alcoholic fatty liver disease |
| Mapp. k36 | 0.14 | 1.29E-02 | 1.00E+00 | Parathyroid hormone synthesis, secretion and action |
| Mapp. k36 | -0.0619 | 9.98E-02 | 1.00E+00 | Parkinson disease |
| Mapp. k36 | 0.0978 | 4.05E-02 | 1.00E+00 | Phospholipase D signaling pathway |
| Mapp. k36 | -0.0682 | 6.56E-02 | 1.00E+00 | Prion disease |
| Mapp. k36 | 0.15 | 5.59E-02 | 1.00E+00 | Regulation of lipolysis in adipocytes |
| Mapp. k36 | 0.0746 | 1.26E-01 | 1.00E+00 | Retrograde endocannabinoid signaling |
| Mapp. k36 | 0.0274 | 6.02E-01 | 1.00E+00 | Thyroid hormone signaling pathway |
| Mapp. k50 | -0.0544 | 4.08E-01 | 1.00E+00 | Arrhythmogenic right ventricular cardiomyopathy |
| Mapp. k50 | 0.0802 | 2.05E-01 | 1.00E+00 | Cardiac muscle contraction |
| Mapp. k50 | -0.0712 | 8.49E-02 | 1.00E+00 | Chemical carcinogenesis - reactive oxygen species |
| Mapp. k50 | 0.016 | 7.62E-01 | 1.00E+00 | Growth hormone synthesis, secretion and action |
| Mapp. k50 | -0.0432 | 2.13E-01 | 1.00E+00 | Huntington disease |
| Mapp. k50 | 0.0284 | 5.47E-01 | 1.00E+00 | Non-alcoholic fatty liver disease |
| Mapp. k50 | 0.0591 | 2.92E-01 | 1.00E+00 | Parathyroid hormone synthesis, secretion and action |
| Mapp. k50 | 0.00922 | 8.06E-01 | 1.00E+00 | Parkinson disease |
| Mapp. k50 | 0.0428 | 3.70E-01 | 1.00E+00 | Phospholipase D signaling pathway |
| Mapp. k50 | -0.0203 | 5.83E-01 | 1.00E+00 | Prion disease |
| Mapp. k50 | 0.0663 | 3.98E-01 | 1.00E+00 | Regulation of lipolysis in adipocytes |
| Mapp. k50 | 0.0385 | 4.29E-01 | 1.00E+00 | Retrograde endocannabinoid signaling |
| Mapp. k50 | 0.207 | 9.22E-04 | 1.06E-01 | Taste transduction |
| Mapp. k50 | 0.00833 | 8.74E-01 | 1.00E+00 | Thyroid hormone signaling pathway |
| No. transcripts | 0.193 | 2.40E-04 | 3.02E-02 | Growth hormone synthesis, secretion and action |
| No. transcripts | 0.14 | 4.26E-05 | 5.59E-03 | Huntington disease |
| No. transcripts | 0.137 | 2.33E-04 | 2.97E-02 | Parkinson disease |
| No. transcripts | 0.212 | 7.06E-06 | 9.75E-04 | Phospholipase D signaling pathway |
| No. transcripts | 0.234 | 6.82E-06 | 9.48E-04 | Thyroid hormone signaling pathway |
| No. transcripts | 0.169 | 9.62E-03 | 9.14E-01 | Arrhythmogenic right ventricular cardiomyopathy |
| No. transcripts | 0.074 | 2.38E-01 | 1.00E+00 | Cardiac muscle contraction |
| No. transcripts | 0.139 | 6.54E-04 | 7.72E-02 | Chemical carcinogenesis - reactive oxygen species |
| No. transcripts | 0.108 | 2.09E-02 | 1.00E+00 | Non-alcoholic fatty liver disease |
| No. transcripts | 0.146 | 8.70E-03 | 8.44E-01 | Parathyroid hormone synthesis, secretion and action |
| No. transcripts | 0.0809 | 2.71E-02 | 1.00E+00 | Prion disease |
| No. transcripts | 0.139 | 7.36E-02 | 1.00E+00 | Regulation of lipolysis in adipocytes |
| No. transcripts | 0.128 | 8.14E-03 | 8.06E-01 | Retrograde endocannabinoid signaling |
| No. transcripts | -0.182 | 3.26E-03 | 3.43E-01 | Taste transduction |
| TIN | -0.273 | 1.54E-05 | 2.08E-03 | Cardiac muscle contraction |
| TIN | 0.167 | 3.82E-04 | 4.70E-02 | Non-alcoholic fatty liver disease |
| TIN | -0.469 | 5.93E-14 | 8.84E-12 | Taste transduction |
| TIN | -0.189 | 3.63E-03 | 3.78E-01 | Arrhythmogenic right ventricular cardiomyopathy |
| TIN | 0.127 | 1.97E-03 | 2.13E-01 | Chemical carcinogenesis - reactive oxygen species |
| TIN | 0.0899 | 8.88E-02 | 1.00E+00 | Growth hormone synthesis, secretion and action |
| TIN | 0.115 | 9.09E-04 | 1.05E-01 | Huntington disease |
| TIN | 0.117 | 3.56E-02 | 1.00E+00 | Parathyroid hormone synthesis, secretion and action |
| TIN | 0.129 | 5.80E-04 | 6.96E-02 | Parkinson disease |
| TIN | 0.105 | 2.55E-02 | 1.00E+00 | Phospholipase D signaling pathway |
| TIN | 0.127 | 5.56E-04 | 6.79E-02 | Prion disease |
| TIN | -0.086 | 2.69E-01 | 1.00E+00 | Regulation of lipolysis in adipocytes |
| TIN | -0.154 | 1.39E-03 | 1.55E-01 | Retrograde endocannabinoid signaling |
| TIN | 0.176 | 7.13E-04 | 8.35E-02 | Thyroid hormone signaling pathway |
| UTR3 length | -0.148 | 2.23E-05 | 2.99E-03 | Huntington disease |
| UTR3 length | -0.183 | 1.21E-06 | 1.70E-04 | Parkinson disease |
| UTR3 length | 0.21 | 1.12E-05 | 1.52E-03 | Phospholipase D signaling pathway |
| UTR3 length | -0.18 | 1.25E-06 | 1.75E-04 | Prion disease |
| UTR3 length | 0.134 | 4.18E-02 | 1.00E+00 | Arrhythmogenic right ventricular cardiomyopathy |
| UTR3 length | -0.19 | 2.78E-03 | 2.95E-01 | Cardiac muscle contraction |
| UTR3 length | -0.115 | 5.64E-03 | 5.75E-01 | Chemical carcinogenesis - reactive oxygen species |
| UTR3 length | 0.13 | 1.46E-02 | 1.00E+00 | Growth hormone synthesis, secretion and action |
| UTR3 length | -0.0891 | 5.90E-02 | 1.00E+00 | Non-alcoholic fatty liver disease |
| UTR3 length | 0.174 | 1.92E-03 | 2.09E-01 | Parathyroid hormone synthesis, secretion and action |
| UTR3 length | 0.159 | 4.37E-02 | 1.00E+00 | Regulation of lipolysis in adipocytes |
| UTR3 length | 0.0892 | 6.85E-02 | 1.00E+00 | Retrograde endocannabinoid signaling |
| UTR3 length | -0.0894 | 1.81E-01 | 1.00E+00 | Taste transduction |
| UTR3 length | 0.164 | 1.83E-03 | 2.04E-01 | Thyroid hormone signaling pathway |
| UTR5 length | 0.191 | 3.62E-04 | 4.53E-02 | Growth hormone synthesis, secretion and action |
| UTR5 length | 0.132 | 4.55E-02 | 1.00E+00 | Arrhythmogenic right ventricular cardiomyopathy |
| UTR5 length | -0.0361 | 5.70E-01 | 1.00E+00 | Cardiac muscle contraction |
| UTR5 length | -0.0657 | 1.13E-01 | 1.00E+00 | Chemical carcinogenesis - reactive oxygen species |
| UTR5 length | -0.0599 | 8.63E-02 | 1.00E+00 | Huntington disease |
| UTR5 length | -0.0741 | 1.16E-01 | 1.00E+00 | Non-alcoholic fatty liver disease |
| UTR5 length | 0.194 | 5.62E-04 | 6.80E-02 | Parathyroid hormone synthesis, secretion and action |
| UTR5 length | -0.0151 | 6.89E-01 | 1.00E+00 | Parkinson disease |
| UTR5 length | 0.135 | 4.85E-03 | 4.99E-01 | Phospholipase D signaling pathway |
| UTR5 length | -0.0466 | 2.08E-01 | 1.00E+00 | Prion disease |
| UTR5 length | 0.209 | 7.85E-03 | 7.85E-01 | Regulation of lipolysis in adipocytes |
| UTR5 length | 0.0351 | 4.72E-01 | 1.00E+00 | Retrograde endocannabinoid signaling |
| UTR5 length | 0.065 | 3.21E-01 | 1.00E+00 | Taste transduction |
| UTR5 length | 0.0967 | 6.62E-02 | 1.00E+00 | Thyroid hormone signaling pathway |
